# A *cis*-Regulatory Mutation of *PDSS2* Causes Silky-Feather in Chickens

**DOI:** 10.1371/journal.pgen.1004576

**Published:** 2014-08-28

**Authors:** Chungang Feng, Yu Gao, Ben Dorshorst, Chi Song, Xiaorong Gu, Qingyuan Li, Jinxiu Li, Tongxin Liu, Carl-Johan Rubin, Yiqiang Zhao, Yanqiang Wang, Jing Fei, Huifang Li, Kuanwei Chen, Hao Qu, Dingming Shu, Chris Ashwell, Yang Da, Leif Andersson, Xiaoxiang Hu, Ning Li

**Affiliations:** 1 State Key Laboratory for Agrobiotechnology, China Agricultural University, Beijing, China; 2 National Engineering Laboratory for Animal Breeding, China Agricultural University, Beijing, China; 3 College of Animal Science and Technology, China Agricultural University, Beijing, China; 4 Science for Life Laboratory, Department of Medical Biochemistry and Microbiology, Uppsala University, Uppsala, Sweden; 5 Department of Animal and Poultry Sciences, Virginia Tech, Blacksburg, Virginia, United States of America; 6 Jiangsu Institute of Poultry Science, Yangzhou, Jiangsu, China; 7 Institute of Animal Science, Guangdong Academy of Agricultural Sciences, Guangzhou, Guangdong, China; 8 Prestage Department of Poultry Science, North Carolina State University, Raleigh, North Carolina, United States of America; 9 Department of Animal Science, University of Minnesota, Saint Paul, Minnesota, United States of America; 10 Department of Animal Breeding and Genetics, Swedish University of Agricultural Sciences, Uppsala, Sweden; 11 College of Animal Science and Technology, Yunnan Agricultural University, Kunming, Yunnan, China; University of Bern, Switzerland

## Abstract

Silky-feather has been selected and fixed in some breeds due to its unique appearance. This phenotype is caused by a single recessive gene (hookless, *h*). Here we map the *silky-feather* locus to chromosome 3 by linkage analysis and subsequently fine-map it to an 18.9 kb interval using the identical by descent (IBD) method. Further analysis reveals that a C to G transversion located upstream of the prenyl (decaprenyl) diphosphate synthase, subunit 2 (*PDSS2*) gene is causing *silky-feather*. All silky-feather birds are homozygous for the G allele. The *silky-feather* mutation significantly decreases the expression of *PDSS2* during feather development *in vivo*. Consistent with the regulatory effect, the C to G transversion is shown to remarkably reduce *PDSS2* promoter activity *in vitro*. We report a new example of feather structure variation associated with a spontaneous mutation and provide new insight into the *PDSS2* function.

## Author Summary

The feather is an excellent model for evolution and development due to its complex structure and vast diversity. Some chickens have silky-feather because of a loss of hooklets in pennaceous feathers, while most chickens have the wild-type normal feather. Hooklets are formed in the last differentiation stage of the life cycle of a pennaceous feather. Chickens with silky-feather are homozygous for a recessive allele (hookless, *h*). Silkie chicken from China is one of the breeds showing the fascinating silky-feather phenotype and the breed has been known for hundreds of years. In this study, we mapped the *silky-feather* locus to an 18.9 kb interval and identified a single nucleotide polymorphism (SNP) completely associated with *silky-feather*. The causative mutation is located 103 base pairs upstream of the coding sequence of prenyl (decaprenyl) diphosphate synthase, subunit 2 (*PDSS2*). The expression of the *PDSS2* gene is decreased in silky-feather skin during feather development *in vivo*. The *silky-feather* allele also reduces the *PDSS2* promoter activity *in vitro*. This is the first report of feather structure variation associated with *PDSS2* and provides new insight into molecular signaling in the late development stage of feather morphogenesis.

## Introduction

The feather is one of the most complex integumentary appendages due to the extensive diversity in shape, size, arrangement and pigmentation, and is therefore an excellent model for evolutionary and developmental biology as variations can occur at each step of development and differentiation. The development of feathers, beginning in the embryo and continuing through several cycles of regeneration after hatching, has been one of the most challenging subjects in the field of avian morphology since the early 19^th^ century [1]. Feathers are clustered in different tracts on the skin and the chicken has about 20 tracts [1]. Feathers from different tracts vary widely and some of the differences are due to different origins of the mesenchyme. The three main types of feathers are contour feathers (pennaceous), down feathers (plumulaceous) and filoplumes [1]. Contour feathers are divided into flight feathers known as remiges and rectrices, and the ordinary body contour feathers. A typical contour feather is composed of the calamus, rachis, barbs and afterfeathers. The barbs have two sets of barbules which are hooked together by the hooklets. Feathers can develop into different types with part or all of the branching structures. Several feather types exist in the domesticated birds and these variations have been selectively bred. Variations in the structure, distribution, length, arrangement and number of feathers are widespread in chickens and pigeons [2]. Factors controlling feather morphogenesis have been studied by poultry geneticists, zoologists and embryologists [1], but only a few spontaneous mutations affecting feather morphogenesis are known. Well-studied natural variations in chicken include the *BMP12* gene that changes the distribution of feathers on the neck (Naked neck trait) [3], the *HOXC8* gene that changes the length of the cranial feathers (Crest trait) [4], and the *KRT75* gene causing a characteristic curled feather rachis and barbs (Frizzle feather trait) [5].

**Figure 1:**
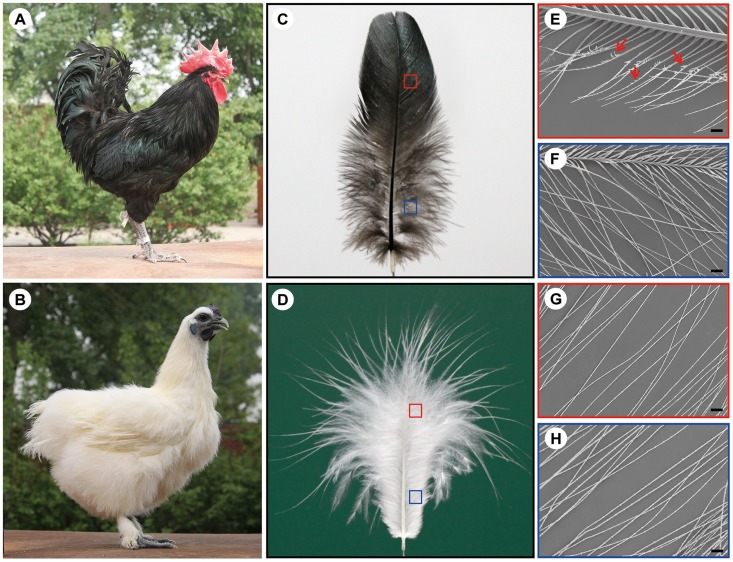
The wild-type and silky-feather phenotype in chickens. (A) Wild-type bird. (B) Silky-feather bird. (C) Wild-type contour feather. (D) Silky-feather contour feather. (E) Pennaceous barbules of wild-type contour feather. (F) Plumulaceous barbules of wild-type contour feather. (G) Pennaceous barbules of silky contour feather. (H) Plumulaceous barbules of silky contour feather. (E–H) Magnification: 100×; scale bar: 100 µm. Schematic magnification of the red and blue boxes in (C) and (D) are shown in (E–H). Arrows in (E) indicate the hooklet. (E) Pennaceous barbules of wild-type contour feather have hooklets, and (G) pennaceous barbules of silky contour feather don't have hooklets.

Variations in contour feather structure usually occur in the distal barbules part of the vane and disturb the interlocking barbules. The structure variations in domesticated birds have been summarized [2], including silky-feathers, frizzle feathers, hypoplasia of tail feathers and henny-feathering, hard and soft feather texture in the chicken; lace-feathering, curled feathers and powder downs, and fat quills in the pigeon; and spiraled feathers in the goose. The silky-feather phenotype is a primary characteristic of the Silkie breed of chicken which is named for this phenotype. The Silkie was first mentioned by Marco Polo in his Asian travelogues in 1298 as “chickens with hair like cats that lay the best of eggs” [6]. Darwin [7] noted that the progeny from matings between Silkie and wild-type chickens did not show the silky-feather phenotype, and a recessive mode of inheritance was subsequently confirmed by Dunn in 1927 [8]. All chickens have similar downy feathers at hatching. The first molting is initiated a few weeks after hatching at which time down feathers are replaced with juvenile pennaceous feathers. In silky-feather chickens beginning with the first pennaceous feathers a clear difference in feather structure is seen as compared to wild-type, with the Silkie chicken maintaining a more downy appearance in the body contour feathers (Figure 1A and 1B). In the closed pennaceous feather portion, wild-type feathers have hooklets on the distal barbules and forms the vane (Figure 1C and 1E) while silky-feathers lack hooklets (Figure 1D and 1G). In the afterfeather portion, both wild-type and silky-feather have the barbules structure but no hooklets (Figure 1F and 1H). The flight feathers and some of the shank feathers can form hooklets in Silkie birds, but fewer than in wild-type chicken (Figure 1B) [9]. This phenotype has apparently been strongly favored during the development of the Silkie breed due to the beautiful fur-like feathers [9]. The Silkie chickens lose the flight function and do poorly in extreme temperatures because of the lack of closed pennaceous vane.

A few variations of feather vane structure are related with hooklets in birds. One variation is the chicken frizzle feather which curves backward and mainly alters rachis structure. The mature frizzle feather structure also exhibits other modifications such as thickening of the barbs and barbules, alteration of the hooklets and other structural abnormalities, however without losing any morphological component [5], [10]. The *frizzle feather* locus is inherited in an autosomal incomplete dominant manner and caused by the *KRT75* gene deletion mutation [5], [10]. Another variation is the silky plumage in pigeons which was also noted by Darwin although he didn't know its inheritance [7]. The structure of the silky plumage (also named *lace-feathering* locus) in domestic pigeons (e.g. Silky Fantail) and Ring-neck Doves is completely different with the silky-feather in chicken [11]. The silky pigeons have hooklets on the barbules, and the hooklets are abnormally thickened [12]. Moreover, the barbules are weak and their elasticity is poor. The *lace-feathering* locus is controlled by an autosomal gene with incomplete dominance [11], [12].

Some signaling molecules involved in feather morphogenesis have been studied, and some feather-branching morphogenesis models are proposed involving the expression of sonic hedgehog (SHH), bone morphogenetic protein (BMP), Noggin, etc. [13]–[18]. SHH is found to mediate the interaction between the epithelium and mesenchyme during feather development [13]. In contrast, BMPs are found to inhibit feather formation [14]. Harris *et al.* [16], [17] suggested that, (i) the activator-inhibitor models of SHH and BMP2 signaling to explain the barb formation, and (ii) an integrated model of feather morphogenesis and evolution to describe the feather branching structures. SHH and BMP2 signaling constitutes a functionally conserved developmental signaling pathway during epidermal appendage development, and the interaction between SHH and BMP2 signaling in feather epithelium has been demonstrated to control the formation of barb ridges and barb variation [16]. Additional inhibitory signal and signal gradient are required for the hierarchical branched structures and feather form (e.g. plumulaceous and pennaceous structures). Variation regulating the SHH and BMP2 may be crucial for the evolution of feather-branching morphogenesis [16], [17]. Yu *et al.* [18] found that the antagonistic interaction between Noggin and BMP4 mediated feather branching, and SHH was required for the formation of barbs. Noggin promotes branching, and BMPs promote rachis formation and inhibit the barb formation. The balance between BMPs and SHH signaling modulate the number and size of the barbs and barb ridges. The interaction of these signals determines their number and fate of marginal plate cells and barbule plate cells, which leads to the feather variants [18]. Previous studies have shown that many signaling molecules are involved in feather morphogenesis, but little is known about the molecular mechanisms of hooklet development and differentiation [1], [19], [20].

In the present study, we show that the silky-feather phenotype is associated with a single regulatory SNP modulating the expression of the *PDSS2* gene.

## Results

### *Silky-feather* is located on chromosome 3

Our initial linkage analysis to map the *silky-feather* locus was based on a genome-wide set of 125 microsatellite markers previously used for mapping growth trait QTLs in the CAU resource population (CAURP) comprised of a Silkie x White Plymouth Rock intercross [21]. The *silky-feather* locus was mapped to the SF01–SF06 (GGA3:69.28–71.63 Mb) interval on chromosome 3 and showed tight linkage with the SF03 marker (70.41 Mb) with a LOD score of 30.1, consistent with the results in another resource population [22]. Refined linkage mapping was done using the Illumina Chicken 60K SNP Beadchip which resulted in the assignment of the *silky-feather* locus to a 380 kb interval between positions 70,201,106–70,581,126 bp (Table 1). This interval contains three genes, Sex comb on midleg-like 4 (*SCML4*), Sine oculis-binding protein (*SOBP*, also named *Jxc1*) and Prenyl (decaprenyl) diphosphate synthase, subunit 2 (*PDSS2*). Refined linkage mapping in another population [22] identified a similar albeit slightly larger location of 70,399,176–70,988,264 bp (about 589 kb) with an overlap of 70,399,176–70,581,126 bp (about 182 kb) from the two populations combined.

**Table 1:** Two-point linkage analysis between *silky-feather* locus and SNP markers on chicken chromosome 3.

SNP Marker^a^	Position (bp)^b^	Recombinant fraction	LOD score
ss317369158	70,119,483	0.01	23.2
rs16294399	70,201,106	0.01	22.9
rs16294517	70,299,532	0	11.1
ss317369395	70,311,973	0	17.8
rs15380516	70,367,159	0	15.1
rs16294597	70,367,382	0	23.2
rs16294606	70,377,047	0	20.2
rs16294615	70,383,717	0	19.6
rs14372495	70,384,172	0	20.2
rs14372520	70,399,176	0	20.5
ss666793689	70,412,158	0	8.4
rs14372560	70,422,653	0	18.4
rs16294682	70,441,580	0	5.7
rs16294701	70,447,648	0	19.6
rs16294719	70,461,007	0	29.2
rs13691298	70,467,968	0	34.0
rs16294744	70,481,788	0	14.8
rs14372652	70,504,365	0	21.7
rs14372671	70,527,941	0	28.0
ss317369621	70,558,349	0	29.5
ss317369629	70,581,126	0.01	31.2
rs10724747	70,640,111	0.01	18.8

a SNP marker contains the Illumina Chicken 60K SNP Beadchip and additional SNP genotyped by SNPlex (Table S1).

b All positions refer to the May 2006 (WUGSC 2.1/galGal3) genome assembly.

### Identical-by-descent (IBD) mapping narrows *silky-feather* to an 18.9 kb interval

We refined the location of the *silky-feather* locus in the CAURP using the identical-by-descent (IBD) mapping method [23]. In the F_0_ generation the White Plymouth Rock is fixed for the *wild-type* allele and the Silkie chicken is fixed for the *silky-feather* allele. The White Plymouth Rock is not suspected of carrying any *silky-feather* haplotype as no introgression between these breeds has occurred to the best of our knowledge. The 38 SNP markers genotyped on 12 White Plymouth Rock and 19 Silkie birds were used for the initial IBD mapping. Of the 38 SNP markers, 20 covered the 380-kb (GGA3:70,201,106–70,581,126) interval without recombination in the linkage analysis (Table 1). Homozygosity for the silky-feather birds was limited to two short haplotype blocks defined by three SNPs respectively: the proximal 57.4-kb (70,384,172–70,441,580) and the distal 56.7-kb (70,447,648–70,504,365) (Figure S1). One White Plymouth Rock bird and some wild-type F_2_ individuals were homozygous for the proximal Silkie shared haplotype, whereas only the silky-feather birds in Silkie and the F_2_ generation were homozygous for the distal Silkie shared haplotype. Therefore, the proximal region was excluded and the *silky-feather* shared haplotype was narrowed to the distal 56.7-kb interval (70,447,648–70,504,365) (Figure S1).

We sequenced one Silkie chicken BAC and one Red Jungle Fowl BAC to identify all sequence polymorphisms associated with the *silky-feather* allele and identified 886 polymorphisms in the 70,349,337–70,487,932 bp interval. An estimated 891 bp gap at 70,470,161–70,471,051 bp in the chicken galGal3 genome assembly was re-sequenced successfully in the Red Jungle Fowl BAC (from the reference chicken genome individual) and was determined to be 626 bp (GenBank KC166241).

We genotyped 34 SNP markers within the 70,441,580–70,484,959 bp interval in 12 breeds for further IBD mapping. This breed panel consisted of 76 samples from Silkie, Kuaida Silky and Lanping Silky chickens all with silky-feather, and 95 samples from nine breeds that have the wild-type phenotype. The upstream IBD boundary (70,460,738 bp) was established based on two heterozygous Silkie and one homozygous Lanping Silky chickens (Table S2). A downstream IBD boundary was not established using this panel. The *silky-feather* haplotype defined in the 70,461,033–70,484,959 bp interval was fixed in all 76 silky-feather chickens (except one heterozygous Silkie bird at 70,466,750 bp) and was not found in the 95 wild-type chickens, indicating that the causative mutation must be located in the 70,460,739–70,504,365 bp region.

**Figure 2:**
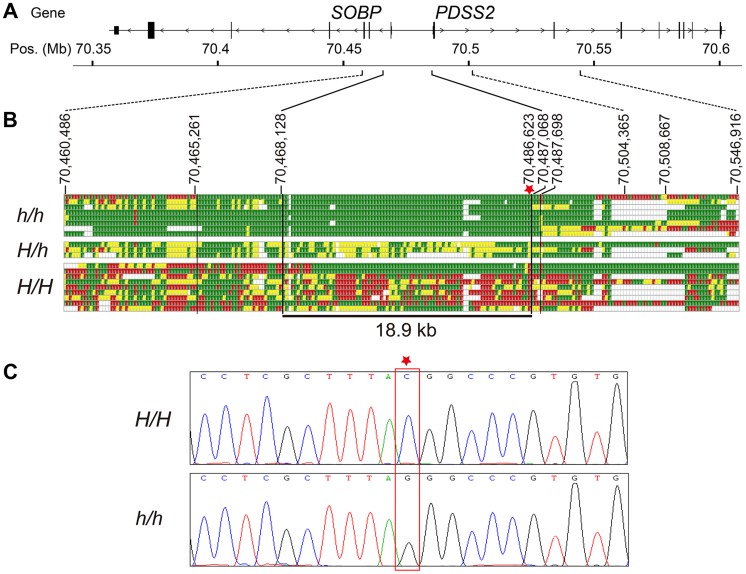
Fine mapping and identifying the causative mutation for *silky-feather*. (A) Schematic structure of *SOBP* and *PDSS2* gene. (B) Identical-by-descent (IBD) mapping of the recessive *silky-feather* (*h*) allele. SNP genotypes for *h/h*, *H/h* and *H/H* individuals are presented. Green and Red: two alternative homozygous genotypes. Yellow: heterozygous genotype. White: missing genotype. The thin black vertical lines are the boundaries defined by the homozygous genotypes in *h/h* birds. The thick black vertical lines are the proximal and distal boundaries of the *h* shared haplotype defined by the heterozygous genotypes. The minimal *silky-feather* shared haplotype is 18.9 kb. Asterisk indicates causative mutation position. (C) Electropherogram represents the DNA sequence across the candidate mutation (marked with an asterisk).

Based on this SNP screening, we selected seven silky-feather birds and nine wild-type birds for re-sequencing. Additionally, three F_1_ heterozygous birds and one F_2_ silky-feather bird were included. The F_2_ bird carried an intact Silkie chromosome and a recombined chromosome within the 70,467,968–70,581,126 bp interval that was of Silkie proximal descent and of White Plymouth Rock distal descent. Interestingly, among the nine wild-type birds, one White Leghorn and one White Plymouth Rock had the identical haplotype with *silky-feather* at 70,472,921–70,484,959 bp (Table S2). We re-sequenced the 49 kb interval (70,460,286–70,509,023 bp) using the Sanger method. Four ∼1.2 kb fragments located within the 70,515,823–70,547,070 bp interval were also re-sequenced. Re-sequencing from the eight silky-feather birds revealed an exclusively s*ilky-feather* shared haplotype at 70,468,129–70,487,067 bp (Figure 2). The upstream and downstream boundaries were identified separately using two different Silkie birds. The 18.9 kb *silky-feather* haplotype was fixed in eight silky-feather birds, and absent in the wild-type birds.

Whole genome sequencing was used to further investigate the location of IBD haplotypes in Silkie chickens in the USA. DNA was pooled from 15 Silkie chickens and was sequenced on the SOLiD platform as described previously [24]. Within the 182 kb region defined from the combined linkage mapping studies a single haplotype was detected as fixed in the Silkies (Figure S2). This haplotype was 21.7 kb (70,467,293–70,489,020 bp) and overlapped perfectly with the 18.9 kb haplotype identified in the Silkie chickens from China.

### Identification of the causative mutation

The causative mutation should be located in this 18.9 kb region, and no structural change in this region was identified in the present study via Sanger sequencing or in our previous study using array CGH [25]. The sequence of the assembly gap in this region (GenBank KC166241) contained 83.5% G and C with numerous poly G, poly C and CpG sites. One SNP and a 9-bp deletion were found within the gap sequence but none was associated with the silky-feather phenotype. Of the other 85 variations, only a C to G transversion at 70,486,623 bp (ss666793747) was perfectly associated with *silky-feather* (Figure 2). The remaining 84 polymorphisms were excluded because at least one *wild-type* (*H/H*) bird showed the same homozygous genotype as *silky-feather* (*h/h*) birds. We genotyped ss666793747 in 718 birds from 33 populations and found that all 337 *silky-feather* chickens were homozygous G/G, 341 *wild-type* chickens were homozygous C/C and 40 known heterozygous birds were G/C (Table 2). These results lead to the hypothesis that ss666793747 is the causal mutation responsible for the silky-feather phenotype.

**Table 2:** Complete association between the *PDSS2(-103C-G)* (ss666793747) mutation and silky-feather phenotype in different populations.

Breed	Genotype		
	G/G	G/C	C/C
**Silky-feather**			
Jinyang Silky	23	0	0
Kuaida Silky	41	0	0
Lanping Silky	16	0	0
Silkie^a^	257	0	0
**Total**	337	0	0
**Heterozygote**			
CAURP F1	0	27	0
Silkie crossbred	0	13	0
**Total**	0	40	0
**Wild-type**			
Aijiao Yellow	0	0	10
Anak	0	0	10
Anyi Gray	0	0	10
Baier Yellow	0	0	10
Beijing You	0	0	20
Bian	0	0	10
Chahua	0	0	10
Chongren Partridge	0	0	10
Dagu	0	0	10
Dongxiang Blue-Eggshell	0	0	10
Gushi	0	0	10
Henan Game	0	0	10
Huiyang Bearded	0	0	20
Jinhu Black-Bone	0	0	10
Langshan	0	0	10
Luyuan	0	0	10
Qingyuan Partridge	0	0	10
Red Jungle Fowl	0	0	36
Shiqiza	0	0	10
Shouguang	0	0	10
Tibetan	0	0	10
Wenchang	0	0	10
White Leghorn	0	0	24
White Rock	0	0	21
Xianju	0	0	10
Xiaoshan	0	0	10
Youxi Partridge	0	0	10
**Total**	0	0	341

a Silkie samples represent three different sub-lines from China.

### Characterization of the *SOBP* and *PDSS2* transcripts

The candidate mutation ss666793747 is located between the *SOBP* and *PDSS2* genes, which are two adjacent genes separated by 16.7 kb. The ss666793747 mutation is 103 bp upstream of the initiator codon ATG of *PDSS2* and 16.6 kb upstream of *SOBP*. Hereafter, we refer to the ss666793747 mutation as *PDSS2(-103C-G)*.

Chicken *SOBP* contains six predicted exons and five introns according to the current annotation of the chicken genome assembly. The coding sequence is well conserved among chicken, human, mouse and other vertebrate species. The human and mouse have a non-coding exon 7. The chicken exon 7 was confirmed by 3′ RACE analysis and the mRNA has been submitted to GenBank with number KC166240. Chicken *PDSS2* contains eight exons and the transcript has been submitted to GenBank with number JX982522. To determine whether *PDSS2(-103C-G)* disrupted the transcription start site (TSS) and the coding sequence of *PDSS2*, we performed 5′ RACE analysis using dorsal skin from three *wild-type* and three *silky-feather* homozygous birds. The most common start site at position −90 (70,486,636 bp) downstream of *PDSS2(-103C-G)* was identical in all the *wild-type* and *silky-feather* birds (Figure S3). Meanwhile, the *PDSS2(-103C-G)* was found to be present in the 5′ UTR in a few clones (Figure S3). Determining the exact TSS of *PDSS2* with 5′ RACE is technically challenging due to the high GC content in this region. Another possible case is that multiple TSSs are located within the small region. The translation start site of *PDSS2* was confirmed and showed no difference between the two genotypes. Exon re-sequencing revealed four synonymous SNPs in *SOBP*, eight synonymous SNPs and a nonsynonymous SNP in *PDSS2*, but none of them was specifically associated with *silky-feather* (Tables S3 and S4).

Expression analysis from postnatal (P) days 60 *H/H* and *h/h* homozygotes by RT-PCR revealed that *PDSS2* and *SOBP* were expressed in all of the tissues for both genotypes (Figure S4). Transcript analysis of *PDSS2* and *SOBP* revealed no splice difference between *H/H* and *h/h* genotypes (Figures S5 and S6).

### Expression analysis indicates *PDSS2* as the causative gene

**Figure 3:**
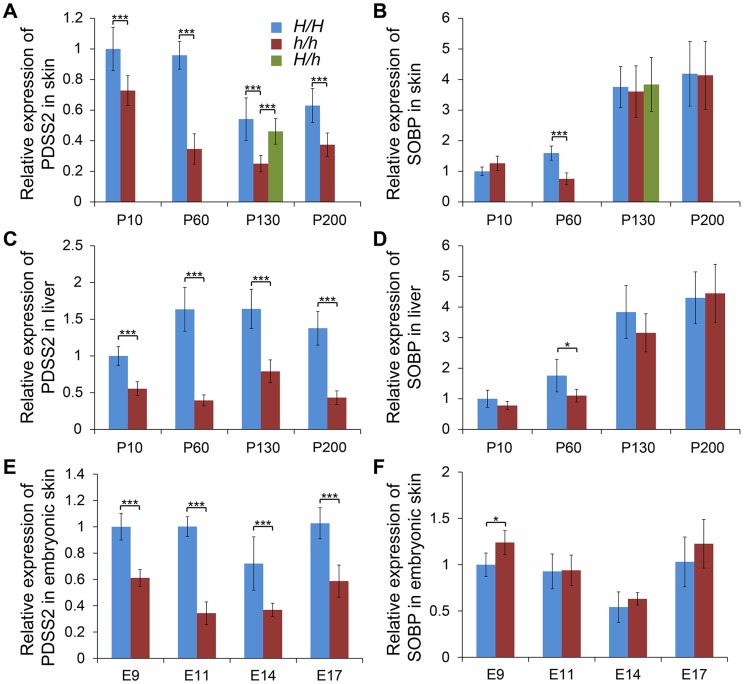
Relative mRNA expression levels of *PDSS2* and *SOBP* in skin and liver. The mRNA expression of (A) *PDSS2* and (B) *SOBP* in postnatal dorsal skin, (C) *PDSS2* and (D) *SOBP* in liver, and (E) *PDSS2* and (F) *SOBP* in embryonic dorsal skin. The mRNA expression is compared with *GAPDH*. *H/H*, *h/h* and *H/h* represent *wild-type* homozygous, *silky-feather* homozygous and heterozygous birds respectively. P10, P60, P130 and P200 represent postnatal (P) days 10, 60, 130 and 200. E9, E11, E14 and E17 represent embryonic (E) days 9, 11, 14 and 17. * indicates P<0.05 and *** indicates P<0.001. The bar represents standard deviation.

We investigated the relative expression levels of *PDSS2* and *SOBP* mRNA in dorsal skin tissue at P10, P60, P130 and P200 from two homozygotes. Quantitative RT-PCR analysis showed that the expression of *PDSS2* was significantly reduced in silky-feather birds at all stages, but *SOBP* expression was only decreased at P60 (Figure 3A and 3B). To explore whether *PDSS2(-103C-G)* is a tissue-specific regulatory mutation, the expression analysis was further performed in liver tissue at these four stages. A similar pattern with reduced *PDSS2* expression in silky-feather birds at all stages and lower *SOBP* expression only at P60 was observed (Figure 3C and 3D), showing that the mutation does not have a tissue-specific effect. Furthermore, the expression of *PDSS2* was decreased significantly in silky-feather birds at embryonic stages, whereas *SOBP* expression was increased at embryonic (E) days 9 (Figure 3E and 3F).

**Figure 4:**
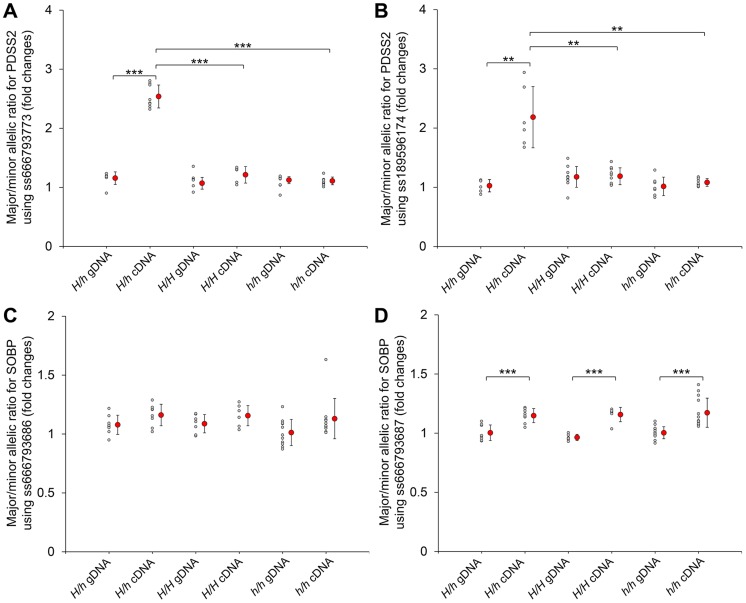
Allelic expression imbalance of *PDSS2* and *SOBP* in skin. The allelic expression imbalance is measured using the pyrosequencing assay. (A and B) The SNPs in the *PDSS2* coding sequence correspond to ss666793773 (exon 8) and ss189596174 (exon 6). (C and D) The SNPs in the *SOBP* coding sequence correspond to ss666793686 (exon 6) and ss666793687 (exon 6). Heterozygous (*H/h*) and homozygous (*H/H* and *h/h*) chickens are tested. The allele with higher expression in cDNA is regarded as the major allele in both genomic DNA (gDNA) and cDNA of the same individual. Small gray circles correspond to the allelic ratio for individual chickens. Large red circles correspond to the average mean value and bar means standard deviation. In homozygous chickens (*H/H* or *h/h*), the ratios of three different stages (P60, P130 and P200) are similar and combined into one group for fewer individuals. The P-value of the difference between gDNAs and cDNAs of the same genotype, and cDNAs of different genotypes is evaluated by a t-test. *** indicates P<0.001. ** indicates P<0.01. In (D), the allelic ratios of cDNA do not have difference among the three genotypes, although they are significantly different from the allelic ratios of gDNA. The difference between the cDNA and gDNA may be due to the SNP signal itself.

We further compared *PDSS2* and *SOBP* expression at P130 in all three genotypes. The expression of *PDSS2* in *H/h* skin was higher than in *h/h* skin, but showed no difference with *H/H* skin (Figure 3A). The expression of *SOBP* in *H/h* birds was similar to *H/H* and *h/h* birds (Figure 3B). An allelic expression imbalance (AEI) analysis demonstrated significantly higher expression of the *H* allele for *PDSS2* in P130 heterozygous *H/h* birds while no such allelic imbalance was observed for *SOBP* (Figure 4). The result implied that *PDSS2(-103C-G)* is a *cis*-acting regulatory mutation, consistent with the expression differences in Figure 3.

We also analyzed the expression in dorsal skin tissue of the other nine genes located in the broad 70,177,177–70,816,125 bp interval. No *SCML4* transcript was detected. Five genes (*BEND3*, *C6orf203*, *QRSL1*, *RTN4IP1* and *ENSGALT00000037260*) were differentially expressed between silky-feather and wild-type birds at P10 and two genes (*OSTM1* and *QRSL1*) at P60 (Figure S7). The expression of *QRSL1* was increased significantly in silky-feather birds at P130 and P200, while the expression of *OSTM1* showed an opposite trend at P200 comparing with P60 (Figure S7). These inconsistent expression patterns in skin suggested that these are more likely to be secondary effects or due to regulatory mutations in linkage disequilibrium with *PDSS2(-103C-G)* rather than long range regulatory effects from *PDSS2(-103C-G)*.

**Figure 5:**
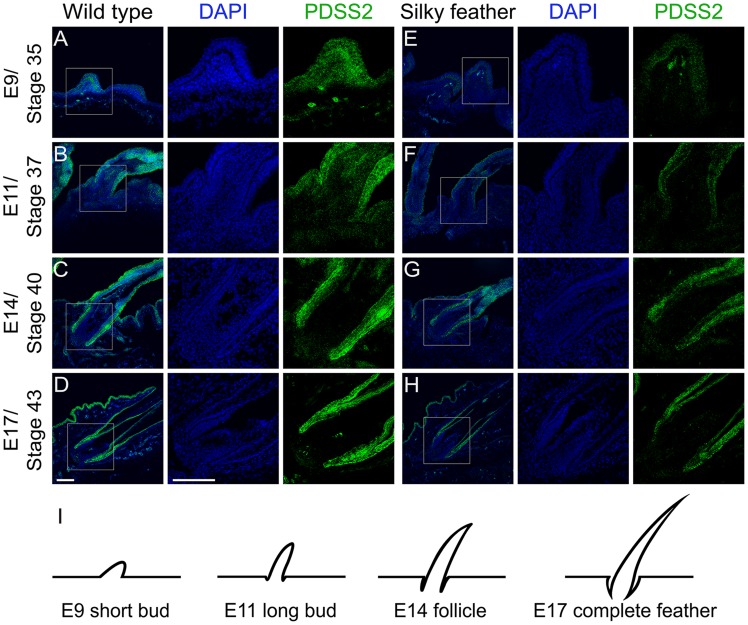
Immunohistochemistry of PDSS2 in wild-type and silky-feather embryonic skins. Sections of wild-type (A–D) and silky-feather (E–H) skins at embryonic (E) days 9, 11, 14 and 17 are immunolabeled with the cPDSS2 antibody. PDSS2 staining is in green, and the nuclei are stained with DAPI (blue). (I) Schematic view of feather development and the sections corresponding to (A–H). The strong green signals that appear obviously unusual in the mesenchyme in (A and E) are suspected to originate from tissue damage caused by the experimental operation (according to bright field and not shown). Scale bar equals 100 µm.

The mRNA expression analysis suggested that silky-feather was likely due to reduced expression of *PDSS2* in the skin. An anti-chicken PDSS2 antibody was custom made and its specificity was confirmed by western blotting and immunofluorescence analysis (Figure S8). The immunofluorescence assay showed that chicken PDSS2 protein is mainly located in the cytoplasm, which is identical with human PDSS2 protein that is localized to cytoplasm according to the Human Protein Atlas (www.proteinatlas.org/ENSG00000164494/tissue) and mitochondrion according to the UniProt database (www.uniprot.org/uniprot/Q86YH6). We performed immunohistochemical staining with the chicken PDSS2 antibody in both *wild-type* and *silky-feather* embryos at day E9, E11, E14 and E17 (Figure 5). PDSS2 expression was observed in feather short bud (Figure 5A and 5E), long bud (Figure 5B and 5F), follicle (Figure 5C and 5G) and complete feather (Figure 5D and 5H). The staining revealed strong PDSS2 expression in feather epithelium and weak expression in distal mesenchyme at E9 (Figure 5A and 5E). At E11 when feather buds elongated, PDSS2 showed a greater intensity in feather epithelium/shaft (Figure 5B and 5F). At E14 when feather follicle formed, PDSS2 was strongly expressed in the collar region of the follicle and sheath (Figure 5C and 5G). At E17 when complete feather formed, striking PDSS2 expression was observed in the proximal follicle and feather sheath (Figure 5D and 5H). The expression of PDSS2 became gradually intense in the skin epithelium (Figure 5). Importantly, the expression of PDSS2 in *silky-feather* embryos was decreased compared with *wild-type* embryos at the same stage, consistent with the results of the mRNA expression (Figure 3E). In addition, the strong expression of PDSS2 in the epidermal cells is consistent with abundant mitochondria in the epidermis of embryonic chick skin [26].

### Luciferase reporter assays demonstrate that the causal mutation reduces *PDSS2* promoter activity

**Figure 6:**
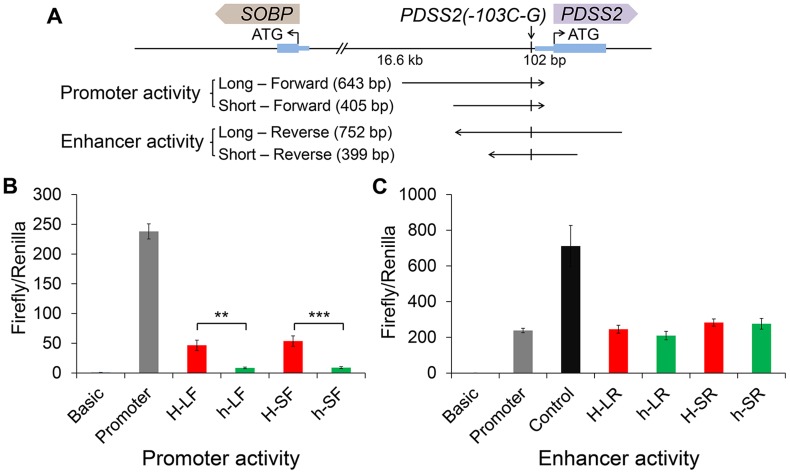
Effects of the *silky-feather* mutation on promoter and enhancer activity using a luciferase reporter assay. (A) Schematic description of vectors used for luciferase reporter assay. Long and short sequences for each allele are used for vector construct separately. The *wild-type* (*H*) or *silky-feather* (*h*) allele forward sequence is inserted to the empty vector pGL3-Basic. The *wild-type* (*H*) or *silky-feather* (*h*) allele reverse sequence is also inserted to the empty vector pGL3-Promoter. (B) Promoter activity and (C) Enhancer activity analyses in chicken DF1 cell are shown for each vector. The pGL3-Basic, pGL3-Promoter and pGL3-Control vectors are used as control. Three technical repeats are performed for each vector in one experiment. Firefly in relation to *Renilla* luciferase levels is calculated with the empty vector pGL3-Basic as reference. The average value of three technical repeats is represented as one activity value. Three separate repeats are performed and used to calculate mean and standard deviation (SD). The activity associated with the *wild-type* (*H*) and *silky-feather* (*h*) constructs are compared using a Student's t-test. ** indicates P<0.01 and *** indicates P<0.001.

We did a BLAST search for the surrounding sequence of *PDSS2(-103C-G)* (30 bp upstream and 30 bp downstream), however, we did not find any significant sequence conservation among birds, reptiles and mammals. Considering the position of *PDSS2(-103C-G)*, we decided to test the promoter effect in “forward” (toward *PDSS2*) orientation and enhancer effect in “reverse” (toward *SOBP*) orientation in the pGL3 luciferase vector (Figure 6A). We transfected chicken fibroblast cells (DF1) in which *PDSS2* and *SOBP* were expressed (Figure S4A), and measured luciferase activity after 24 hours. Two different length fragments were used separately. Compared to the promoterless vector, the *wild-type* vectors increased luciferase activity ∼46-fold (long, H-LF) and ∼53-fold (short, H-SF) whereas the *silky-feather* vectors increased the activity only ∼8-fold (long, h-LF) and ∼9-fold (short, h-SF) (Figure 6B). Thus, the two constructs had promoter activity and most importantly, the *silky-feather* construct decreased promoter activity compared to *wild-type* consistent with the observed differences between alleles *in vivo* (Figure 3A, 3C and 3E). No enhancer activity was detected in reverse direction in *wild-type* or *silky-feather* (Figure 6C). The Abd-B factor was predicted to bind to *wild-type* promoter with score 86.6 (transcriptional factor search, TF2 SEARCH) but not the *silky-feather* allele [27], which might be responsible for the difference in promoter activity.

## Discussion

Silky-feather is a recessive Mendelian trait found in only a few chicken breeds that dramatically alters the structure and appearance of the juvenile and adult chicken feather. We herein show that a single-base change upstream of *PDSS2* is responsible for silky-feather in chicken based on high-resolution mapping and expression analysis. We mapped the *silky-feather* locus to a 182 kb region by linkage analysis using two separate mapping populations and then fine mapped it to an 18.9 kb region using IBD mapping. The identification of an overlapping 21.7 kb IBD region in a second population of Silkie chickens is further indication that this region harbours the causal mutation. Within the minimal haplotype region, SNP *PDSS2(-103C-G)* was completely associated with *silky-feather*. Of the two flanking genes, *PDSS2* showed differential expression in both skin and liver at all stages while *SOBP* only showed differential expression at P60 and E9. Furthermore, consistent with this finding, the *silky-feather* allele was associated with reduced promoter activity in a luciferase assay. We conclude that *PDSS2(-103C-G)* is a *cis*-acting regulatory mutation that causes altered expression of *PDSS2* and is the primary cause for the development of the silky-feather phenotype. We should note that we cannot exclude the possibility that (i) this mutation in the 5′ UTR of *PDSS2* can influence gene expression at the level of translation via other mechanisms such as translation efficiency and mRNA stability [28], [29], and (ii) differential expression of *SOBP* [30] and/or other genes at some specific stage may have some impact on feather (or hooklet) development.

Among silky-feather chicken types, Kuaida Silky chicken was developed by crossing Silkie with Broiler, inheriting the *silky-feather* haplotype from the Silkie. To our knowledge, there is no gene introgression between Jinyang Silky or Lanping Silky and the Silkie breed, and both of these breeds share no other phenotypic characters with Silkie. However, both of them share the 18.9 kb IBD *silky-feather* haplotype with Silkie. Thus, the same ancestral *silky-feather* mutation most likely originated before the formation of these breeds.

The interactions between epithelium and mesenchyme play a critical role in the formation and development of feathers [19]. The epithelium over feather tract has potential for forming the feather field and transforms into individual feather primordia through an activator-inhibitor mechanism [15], [17]. The competition between signaling activators (e.g. SHH) and signaling inhibitors (e.g. BMP) results in the local expression of genes (e.g. SHH) in the feather primordium [13], [14], [17]. This inductive process resides in the mesenchyme and is also epithelium-dependent [19]. Then feather bud forms with an anterior-posterior orientation involving inductive signals, e.g. the expression of Wnt7a in the posterior bud epithelium [31]. In the late long-bud stage, the feather follicle begins to form in the dermis. Subsequently, the natal down feathers and adult alternative feathers with branches emerge from follicles. The immunohistochemistry showed that PDSS2 was persistently expressed in the feather epithelium, mesenchyme, follicle and sheath in both the *wild-type* and *silky-feather* chickens (Figure 5). The gradually enhanced expression of PDSS2 coinciding with feather development indicates that it plays an important role in the formation of the feather, perhaps together with other signals from the follicle.

Feather hooklets are formed in the last differentiation stage of the life cycle of a pennaceous feather. The feather pulp is filled with mesenchymal tissues transiently, and the branch is formed through differential cell death [32]. The different parts of the feather involve different keratin proteins, the presence or absence of which is associated with several mutant feather phenotypes [33], [34]. The formation of hooklets are primarily due to an increased number of barbule cells [35], [36], which can grow into hooklets after differentiation and keratinization [37]. Wedge cells, which are supportive cells of regenerating adult feathers, accumulate periderm granules and corneous material, and then degenerate gradually to produce hooklets that allow individual barbs to align and form a closed vane in mature pennaceous feathers [37], [38]. The expansion of the feather β-keratins genes is thought to have contributed to the evolution and diversity of feathers [39]. An uncharacterized feather β-keratin gene, named *barbule specific keratin 1* (*BLSK1*), is expressed specifically in feather follicles that generate pennaceous barbules but not in follicles that generated plumulaceous barbule [40]. Further expression experiments indicated that *BLSK1* might be involved in the formation of pennaceous barbules and hooklets. In silky-feathers, no hooklets form in apical cells of barbules in plumulaceous or pennaceous feathers. The feather follicle forms at the embryonic stage while contour feathers form after the first molting. During development, silky-feathers lack hooklets in pennaceous feathers compared with the wild-type. The expression of *PDSS2* was decreased in silky-feather dorsal skin and feathers during all stages (Figures 3 and 5). We hypothesize that the persistently decreased expression of *PDSS2* changes barbule cell differentiation and spatial reorganization in the feather follicle and results in the lack of hooklets in silky-feathers.

The theory of feather morphogenesis hypothesized a hierarchical series of stages characterized by successive evolution of tubularity, barbs, the rachis, barbules and hooklets resulting in the closed pennaceous vane [20], [41], [42]. Diversification of feather complexity and function is achieved by independence, covariation and interaction among plumage modules [42]. Barbule plate cells were hypothesized to mature earlier than the central ramus cells for the nutrient transport [43]. Microscopic and ultrastructural analyses of feathers have been focused on the barbs and barbules, but few on cell composition [1], [42], [44]. Electron microscopy has revealed some processes of cell differentiation within developing feathers, but the formation of barb ridges and the cell junctions between barb and barbule cells are nearly unknown and will be an essential area of feather biology research [36], [37]. In contrast to the *frizzle* mutation that affects rachis and barb morphogenesis [5], *silky-feather* is the first locus characterized at the molecular level that affects hooklet morphogenesis. Since the hooklets are uniquely absent in the pennaceous feathers of silky-feather chickens across all the birds, it is difficult to infer the exact role of the *PDSS2* gene in the development and evolution of feathers from the molecular evidence presented in this study. Further studies will provide clues into whether the *PDSS2* is involved in the formation of barbules and hooklets in different feather tracts of more species.

*PDSS2* encodes a prenyl diphosphate synthase that is essential for ubiquinone (Coenzyme Q) biosynthesis, which is required for mitochondrial respiratory electron transport. In human, missense mutations in *PDSS2* were reported to cause Coenzyme Q_10_ (CoQ_10_) deficiency with Leigh syndrome with nephropathy [45]. In the patient's skin fibroblasts, CoQ_10_ decreased to 12% of control cells and ATP synthesis decreased by 51% compared with the control [46]. Similar kidney disease was seen in mice carrying *PDSS2* missense mutations and in glomerular podocytes conditional *PDSS2* knockout mice, but not in renal tubular epithelium, monocytes, or hepatocytes conditional knockout mice [47]. In homozygous missense mutant *PDSS2* mice, CoQ content in the kidney was significantly lower compared with that in wild-type mice [47], [48]. Although these *PDSS2* missense mutant mice showed mitochondrial respiratory chain deficiency in all organs studied (brain, kidney, liver and muscle), another study showed that only the affected kidney organs showed increased reactive oxygen species (ROS) production and oxidative stress [49]. All of the results indicated that the tissue-specific *PDSS2* dysfunction associated with CoQ deficiency might be responsible for the renal disease phenotype [47]–[49]. Cerebellum dysfunction is often associated with ubiquinone deficiency and consistent with that cerebellum hypoplasia and cerebellar ataxia were observed in conditional knockout mice due to increased ectopic apoptosis [50].

Despite *PDSS2* having an important role in CoQ biosynthesis and abnormal *PDSS2* expression initiating kidney and nervous system problems in human and mouse, little is known about the effect of *PDSS2* mutations in the skin. In this study, we showed that lower expression of *PDSS2* in silky-feather chicken skin affected feather development. Mice with keratinocyte-specific deficiency in mitochondrial transcription factor A had an abnormally thick epidermis, lacked hair and showed defects in differentiation [51]. Further analysis showed that the keratinocytes did not produce mitochondrial ROS, which resulted in the impaired Notch and β-catenin signaling pathways during skin development. The increased apoptosis in hair follicles and hair loss in these mice should be caused by the impaired Notch signaling in epidermal differentiation and β-catenin signaling in hair follicle growth. These findings revealed that the mitochondrial ROS influenced the keratinocyte differentiation and hair follicle development [51]. Except for the silky-feather phenotype, none of the QTLs controlling growth, body composition or body size traits is co-localized with the *PDSS2* locus in the same pedigree, indicating that the *silky-feather* mutation has no pleiotropic effect on these quantitative traits [21], [52]–[54].

Silkie skin transplantation experiments have indicated that the determining factors for hooklet development reside in the feather follicle [55], indicating that the mechanism of the *silky-feather* mutation in the feather follicle is independent of the endocrine status. However, some similar but non-genetic silky-feather phenotypes are known. Similar long silky-feathers were first observed in thyroidectomized Brown Leghorn chickens in 1927 [56], and later in hypothyroid White Leghorn chickens [57]. At the same time, hypothyroid chickens are usually small, obese with increased abdominal fat, and have a small, dry comb, which are different from Silkie fowl. Silky-like feathers can develop in normal chickens with excessive dosages of thyroid [58]. Furthermore, the silky-feather barbs in Silkie chickens can form barbules and hooklets after regional depluming and a subcutaneous injection of thyroxine [59], [60]. The thyroid hormone, together with other hormones, are known to contribute to specific aspects of hair growth [61]. Those experiments revealed that the thyroid hormone played an important role in the feather development, which was also found to influence the rate of feather regeneration and the number of regenerated papillae in the Red-headed Buntings (*Emberiza bruniceps*) [62]. Thyroid hormone has been well reviewed for the effect on cellular respiration through changing expression of respiratory genes and modulation of inner membrane structure [63]. Some of the nuclear encoded respiratory genes mRNA appears to be induced by thyroid hormone. Considering that thyroidectomized and hypothyroid wild-type chicken lost the hooklet and compensatory thyroid hormone in Silkie birds formed hooklet, the hooklet morphogenesis might be dependent on normal mitochondrial respiratory function affected by the presence of thyroid hormone. In silky-feather chicken skin, lower expression of *PDSS2* may decrease the CoQ_10_ synthesis, which influences the mitochondrial respiratory chain in the feather follicle.

The evolutionary origin of feathers has been under debate for more than 150 years [41], [43], [64]. Numerous feather structures have been found in dinosaur fossils that have improved our understanding of the origin and evolution of this highly branched epidermal structure [41], [43], [64]. Pennaceous feathers found in dinosaurs demonstrated that modern feathers evolved in non-avian dinosaurs and their arrangement and distribution provided new insight into the dinosaurian hypothesis of bird origins [65]. The simple pennaceous feathers of *Protarchaeopteryx* showed that remiges and rectrices evolved earlier than flight in theropod dinosaurs [66], with variation in feather size and possible color appearing later in *Anchiornis* [67]. By understanding the process by which feathers grow and develop, we can gain insight into how this highly branched epidermal structure may have evolved. Here we describe a mutation in the modern day pennaceous feather that affects a portion of the feather that is crucial to flight. Further studies on how *PDSS2* contributes to normal hooklet morphogenesis may provide us with a better understanding of the origin and evolution of pennaceous feathers.

The *PDSS2(-103C-G)* mutation is presumed to alter the interaction with one or more transcription factors. Regulatory mutations are an important contributor to phenotypic diversity, but it is challenging to establish genotype-phenotype relationships for regulatory mutations and convincingly prove causality [68], [69]. However, a number of *cis*-regulatory element (CRE) mutations have been found to underlie phenotypic variation in domestic animals and CREs can regulate cell-type-specific expression of genes [69], [70]. For example, regulatory mutations with phenotypic effects in chicken include: regulatory mutations in *BCDO2* associated with yellow skin [71], copy number variation in intron 1 of *SOX5* with Pea-comb [72], a deletion of an enhancer element upstream of *SOX10* underlies the Dark brown plumage phenotype [73], a large insertion downstream of *BMP12* causes Naked neck [3], a complex rearrangement involving *EDN3* causes fibromelanosis [24], an inversion resulting in a genomic relocalization of *MNR2* causes Rose-comb [74] and an *EAV-HP* insertion in *SLCO1B3* causes Blue-eggshell [75], [76]. In addition to these regulatory mutations representing structural changes, we now provide a single base nucleotide substitution in non-coding DNA underlying a monogenic trait in chicken.

## Materials and Methods

### Ethics statement

All the chickens were fed and handled according to relevant national and international guidelines.

### Animals

The China Agricultural University Resource Population (CAURP) consisted of 31 F_0_, 19 F_1_ and 229 F_2_ individuals that were used for linkage analysis. The F_0_ generation consisted of 12 White Plymouth Rock chickens and 19 Silkie chickens homozygous for the *wild-type* and *silky-feather* alleles, respectively. All 19 F_1_ birds showed the wild-type phenotype. The observed ratio of 162 wild-type birds and 67 silky-feather birds among the 229 F_2_ birds, did not deviate significantly from the expected 3∶1 ratio (χ^2^ = 2.21 for 1 df, P>0.05). The USA population used for linkage analysis was derived from a single New Hampshire breed male, homozygous *wild-type*, and a single Silkie female chicken, homozygous for the *silky-feather* allele. A single male F_1_ individual was mated to 10 Silkie females and 180 backcross progeny were used for linkage mapping as previously described [22].

The majority of the DNA samples used for IBD analysis and putative causal mutation genotyping were from Jiangsu Institute of Poultry Science. Additional Silkie samples were from Guangdong and Beijing, China; North Carolina and Wisconsin, USA.

### Linkage analysis

Genotyping of the CAURP was performed with 125 microsatellite markers and the Illumina Chicken 60K SNP Beadchip [21], [53], [77]. Eight microsatellite markers were genotyped using ABI 3100 DNA Genetic Analyzer (Applied Biosystems) (See Table S5 for marker information). An additional 16 SNPs were genotyped using the SNPlex genotyping system (See Table S1 for SNP information). Linkage analysis was performed using the CRIMAP software [78]. The USA population was genotyped for 30 markers within the region previously reported [22] using a custom GoldenGate BeadXpress SNP panel.

### Identical-by-descent (IBD) mapping and re-sequencing

Two BAC clones were resequenced. The RJF BAC CH261-103J18 spanning 70,324,632–70,520,054 bp on chromosome 3 was from BACPAC Resource Center (BPRC), and the Silkie BAC 292C7 spanning 70,349,337–70,487,932 bp was identified by PCR from a Silkie chicken BAC library [79]. BAC DNA was sheared to target sizes from 1.5 kb to 3 kb and cloned into pUC118 vector. The sub-clones were sequenced using an Applied Biosystems 3730xl DNA Analyzer. The RJF genome sequence galGal3, assembled by the Washington University Genome Sequencing Center (WUGSC), was used as the reference for alignment. The RJF-BAC was accomplished with 7.4-fold genome coverage and the Silkie BAC with 8.5-fold coverage.

IBD mapping was performed using a panel of 12 breeds (Table S2 for details). This included 76 silky-feather individuals of Silkie, Kuaida Silky and Lanping Silky, all homozygous *h/h*, and 95 wild-type birds (*H/H*) representing nine breeds. The samples of Silkie chickens originated from Beijing, Yangzhou and Guangzhou, China. The Kuaida Silky population was generated by crossing Silkie and a commercial Broiler line, followed by selection for Silkie-like appearance and rapid growth for five generations. Lanping Silky originally came from Yunnan, China, and there was no genetic introgression with Silkie as far as we knew. SNPs were genotyped using the Sequenom MassARRAY platform (see Table S1 for SNP information).

Based on the SNP genotyping information, eight silky-feather birds, nine wild-type birds and three heterozygous birds were used for re-sequencing. Primers were designed for overlapping fragments (primer sequence information in Table S5). The PCR amplicons were purified and directly sequenced using ABI 3730xl DNA Analyzer (Applied Biosystems). DNA sequences were analyzed using DNASTAR software (DNASTAR).

### Pyrosequencing

Genotyping of *PDSS2(-103C-G)* (ss666793747) and ss666793770 was performed using pyrosequencing. The pyrosequencing PCR assays contained 40 ng gDNA, 1× PCR buffer, 1× Q-solution (Qiagen), 200 µM dNTP, 400 nM of each primer, 2 IU LongAmp Taq (NEB), and H_2_O was added to achieve a final volume of 25 µl. A touchdown PCR protocol was used, including 94°C for 5 min, 10 cycles of 94°C, 63°C (−1.0°C/cycle), and 65°C for 30 s each, followed by 30 cycles of 94°C, 53°C, and 65°C for 30 s each, and a final extension at 65°C for 5 min. The PCR product was analyzed by 2% agarose gel electrophoresis and used for pyrosequencing according to standard protocol of PyroMark ID (Qiagen).

### Expression analysis

Tissues used for expression analysis were from Silkie (*h/h*) birds at postnatal (P) days P10 (n = 7), P60 (n = 7), P130 (n = 10), P200 (n = 10) and White Leghorn (*H/H*) birds at P10 (n = 7), P60 (n = 7), Beijing You (*H/H*) at P130 (n = 10), Huiyang Bearded (*H/H*) birds at P200 (n = 10). Heterozygous (*H/h*) birds at P130 (n = 11) used for allelic expression imbalance originated from a cross between Silkie (*h/h*) and Youxi Partridge (*H/H*) chicken. Dorsal skin and liver were collected and stored in liquid nitrogen. Embryonic dorsal skin tissue was collected from Silkie (*h/h*) and White Leghorn (*H/H*) birds. Samples from five birds of each genotype were collected at embryonic (E) days 9, 11, 14 and 17. Tissue was homogenized using TissueLyser LT (Qiagen). Total RNA was extracted using TriZol (Ambion). 1 µg total RNA was used in the first strand cDNA synthesis (Promega) with oligo (dT) 18 primers. The details of primer sequence used for RT-PCR were presented in Table S5.

The 5′ rapid amplification of cDNA ends (RACE) was performed with the 5′ RACE System for Rapid Amplification of cDNA Ends, Version 2.0 (Invitrogen). RNA was extracted from three Beijing You (*H/H*) and three Silkie (*h/h*) chicken dorsal skin tissues at P100 using Trizol reagent (Ambion). The RNA was treated with DNase I (Qiagen) and purified using RNeasy Mini Kit (Qiagen). 3 µg RNA for each sample was used for reverse transcription in a 25 µl reaction with SuperScript III (Invitrogen), which was incubated at 55°C for 50 min for the high GC content. The original and the nested PCR assays contained 3 µl dC-tailed cDNA (or 1∶100 dilution of primary PCR product), 1× PCR buffer, , 1× Q-solution (Qiagen), 5% DMSO, 400 µM dNTP, 400 nM of each primer, 2.5 IU LongAmp Taq (NEB), H_2_O was added to give a final volume of 25 µl. A touchdown PCR protocol was used, including 95°C for 3 min, 15 cycles of 95°C for 20 s, 65°C (−1.0°C/cycle) for 30 s, and 65°C for 2 min, followed by 20 cycles of 95°C for 20 s, 50°C for 30 s, and 65°C for 2 min, and a final extension at 65°C for 5 min. The PCR fragments were purified and sub-cloned into the pMD19-T vector. At least 16 clones per sample were selected for sequencing. Primers used for 5′ RACE were presented in Table S5.

Quantitative real-time PCR were performed with triplicate on LightCycler 480 (Roche). *PDSS2* gene was detected using 40 ng cDNA, 1× Taqman Master mix (Applied Biosystems), 750 nM of each primer and 250 nM probe in a total volume of 20 µl with a PCR condition of 15 min at 95°C and 40 cycles of 95°C for 15 s and 60°C for 1 min. For analysis of *SOBP*, *OSTM1*, *SEC63*, *BEND3*, *C3H6orf203*, *QRSL1*, *RTN4IP1*, *AIM1*, and *ENSGALT00000037260*, 40 ng cDNA, 1× SYBR green mix (Applied Biosystems) and 300 nM of each primer were used in a total volume of 20 µl with a PCR condition of 15 min at 95°C and 40 cycles of 95°C for 15 s and 60°C for 1 min, followed by a melting curve analysis. Expression level data was normalized using *GAPDH* as endogenous reference gene and calculated using the 2^−ΔΔCt^ method. Student's T-test was used to compare different groups.

### Allelic expression imbalance analysis

Pyrosequencing was used to test allelic expression imbalance of *PDSS2* and *SOBP*. Total RNA was purified with DNase I (NEB) and minus-RT (not reverse transcribed) PCR products were used as negative controls. Two SNPs in *PDSS2* (ss666793773 in exon 8 and ss189596174 in exon 6) and two SNPs in *SOBP* (ss666793686 and ss666793687 in exon 6) were designed for PCR amplification. The pyrosequencing PCR assays contained 40 ng cDNA (or gDNA), 1× PCR buffer, 200 µM dNTP, 400 nM of each primer, 2 IU LongAmp Taq (NEB), H_2_O was added to give a final volume of 25 µl. A touchdown PCR protocol was used for the pyrosequencing SNP genotyping test including 94°C for 5 min, 10 cycles of 94°C, 63°C (−1.0°C/cycle), and 65°C for 30 s each, followed by 30 cycles of 94°C, 53°C, and 65°C for 30 s each, and a final extension at 65°C for 5 min. The PCR product was analyzed by 2% agarose gel electrophoresis and used for pyrosequencing according to standard protocol of PyroMark ID (Qiagen). The relative proportion of each allele was obtained using the AQ analysis mode (allele quantification). The details of all primer sequences were given in Table S5.

### Cell culture and transient transfection

DF1 (chicken fibroblast cell line) and 293T (human embryonic kidney cell line) cells were cultured at 37°C in a 5% CO_2_ atmosphere in Dulbecco's Modified Eagle's Medium (DMEM) containing 4.5 g/l of glucose (Gibco) and supplemented with 10% fetal bovine serum (FBS).

The enhanced green fluorescent protein (eGFP) was fused to the N-terminus of the chicken *PDSS2* (*cPDSS2*) gene by fusion PCR. The eGFP-cPDSS2 was cloned into pcDNA3.1(+) using *BamHI* and *XhoI* sites and used for transfection. 293T and DF1 cells were transiently transfected using FuGENE HD Transfection Reagent (Promega) according to the technical manual.

### Antibody and western blotting

An anti-cPDSS2 monoclonal antibody was custom made in mouse using the immunizing peptide IGISTWKEQV-amide corresponding to amino acid residues 221–230 (Abmart, Shanghai, China).

Cells were collected at 48 h after transfection. Protein was extracted from cells with Cell Lysis Buffer (Beyotime). The proteins (30 µg of total cell protein per lane) were separated by 12% SDS-PAGE and transferred to polyvinylidene difluoride (PVDF) membranes according to standard protocols. The membranes were blocked and incubated with anti-cPDSS2 (1∶1000), anti-GFP (1∶1000, ab6556, Abcam) and anti-alpha tubulin (1∶1000, sc-53646, Santa Cruz) antibodies overnight at 4°C. The Membranes were subsequently incubated with horseradish peroxidase (HRP) conjugated goat anti-mouse or goat anti-rabbit secondary antibodies (1∶10000) and visualized using SuperSignal West Dura Extended Duration Substrate (Thermo Scientific).

### Immunofluorescence

293T and DF1 cells (transient transfection and wild-type control) were cultured on poly-lysine-coated coverslips for 36 h before staining. Cells were fixed in 4% paraformaldehyde for 15 min at room temperature, permeabilized with 0.3% Triton X-100 for 10 min, and blocked for 1 h in blocking solution (2% goat serum, 1% BSA, 0.1% Triton-X and 0.05% Tween 20 in PBS). Cells were incubated with cPDSS2 antibody (1∶50) overnight at 4°C. Cells were washed three times with PBS and incubated with Alexa Fluor 594 Goat Anti-Mouse IgG Antibody (1∶500, Invitrogen) for 1 h at room temperature. Cells were washed three times with PBS and treated with DAPI (1 µg/mL) for 5 min. The slides were washed with PBS, mounted with Antifade mounting medium (Beyotime) and imaged with the Olympus Fluoview FV1000 confocal microscope. Images were formatted, resized, enhanced and arranged using FV10-ASW and Adobe Photoshop.

### Immunohistochemistry analysis

The embryonic dorsal skin tissues were fixed in 4% paraformaldehyde in PBS for 3 h, followed by 15 min wash with PBS and incubated in 30% sucrose overnight at 4°C. Tissues were frozen in OCT (Sakura) and sectioned to obtain 10 µm thickness. The sections were rehydrated and blocked for 2 h in blocking solution (2% goat serum, 1% BSA, 0.1% Triton-X and 0.05% Tween 20 in PBS). cPDSS2 antibody was diluted 1∶100 in blocking solution and incubated overnight at 4°C. The sections were washed three times for 5 min in PBS and incubated with Alexa Fluor 488 Goat Anti-Mouse IgG Antibody (1∶400, Invitrogen) for 2 h at room temperature. The nuclei were stained with DAPI. Samples were analyzed using Olympus Fluoview FV1000 confocal microscope with the same parameters. Images were processed using FV10-ASW and Adobe Photoshop.

### Luciferase reporter analysis

Two fragments containing *PDSS2(-103C-G)* were generated with PCR and cloned into pGL3 Basic vector (Promega), a longer 643 bp fragment (70,486,018–70,486,660 bp) and a shorter 405 bp fragment (70,486,256–70,486,660 bp) in the “forward” (toward *PDSS2*) orientation. A longer 752 bp fragment (70,486,339–70,487,090 bp) and a shorter 399 bp fragment (70,486,444–70,486,842 bp) in the “reverse” (toward *SOBP*) orientation were also amplified and cloned into pGL3 Promoter vector (Promega). *NheI* and *XhoI* sites were selected to construct the vector. DF1 cells plated on 24 wells were transfected at 70–80% confluency with 720 ng of the pGL3 reporter plasmid and 80 ng of pRL-TK Renilla luciferase construct by 2 µl Lipofectamine 2000 (Invitrogen) for each well. The luciferase activity was measured 23–24 h after transfection using the Dual-Glo Luciferase Assay System (Promega) and an Infinite F200 Luminometer (Tecan, Switzerland). Ratios of firefly luminescence/*Renilla* luminescence were calculated, and normalized to control samples (Basic vector). For each test construct, one expression value was the average of three technical replicates in each plate and three separate operations were carried out to represent the final value. The pGL3 Basic vector, pGL3 Promoter vector and pGL3 Control vector were used as control.

### URL

Information on the chicken genome sequence is available at http://www.genome.ucsc.edu (May 2006, WUGSC 2.1/galGal3).

### Accession numbers

The sequence data presented in this paper have been submitted to GenBank with accession numbers KC166240, KC166241, JX982522 and JX982523.

## Supporting Information

Figure S1 IBD mapping in CAURP narrows the *silky-feather* locus to 56.7 kb interval. The genotypes of 38 SNP markers covering about 770 kb interval (70,006,815–70,776,317 bp) in F_0_ individuals are shown. The *silky-feather* is mapped to the 380-kb interval (70,201,106–70,581,126 bp) by linkage analysis. *H/H* represents *wild-type* homozygotes and *h/h* represents *silky-feather* allele homozygotes. Red and Green: two alternative homozygous genotypes. Yellow: heterozygous genotype. White: missing genotype. Homozygosity for the Silkie birds is limited to two short haplotype blocks marked by black thick lines respectively: the proximal 57.4-kb (70,384,172–70,441,580 bp) and the distal 56.7-kb (70,447,648–70,504,365 bp). The distal block is exclusively shared by Silkie birds.
(TIF)

Figure S2 IBD haplotype detected by whole genome sequencing in Silkies from USA. A single extended genomic region fixed for the variant allele at all detected SNPs and representing an IBD haplotype is found in a pool of 15 Silkie chickens from the USA by whole genome sequencing. Variant allele frequency (left y-axis) is indicated in blue and coverage (right y-axis) is indicated in red. The IBD haplotype in the USA Silkies is 21.7 kb (70,467,293–70,489,020 bp) and completely overlaps the 18.9 kb IBD haplotype identified in Silkies from China.
(TIF)

Figure S3 The 5′ RACE analysis of *PDSS2* in skin tissue. Figure shows the DNA, mRNA and protein information of part of *PDSS2* in skin. Three wild-type (*H/H*) and three silky-feather (*h/h*) dorsal skin tissues are used for 5′ RACE analysis as described in Materials and Methods. The total number of random RACE clones for each sample is included in the bracket. The 5′ ends of clones are indicated by the vertical lines with Arabic numerals to indicate the number of clones isolated for each site. The A of the translation start site (ATG) is defined as position +1. The nucleotide positions relative to the ATG are marked with solid circle under each site. The causative mutation *PDSS2(-103C-G)* (70,486,623 bp) is highlighted in uppercase and indicated by the red arrow. The most common start site (70,486,636 bp) is highlighted in bold and indicated by the black arrow. Most of the 5′ ends are located between position −81 and −96 around the most common position −90. Specifically, the proportions from all the six samples are 86.4% (19/22, HH-01), 72.2% (13/18, HH-02), 84.2% (16/19, HH-03), 100% (20/20, hh-01), 87.5% (14/16, hh-02) and 100% (17/17, hh-03), respectively. Furthermore, *PDSS2(-103C-G)* is contained in a few clones. Determining the exact transcription start site of *PDSS2* with 5′ RACE is technically challenging due to the high GC content (75.8%) in 339-bp sequence of the presumed 5′ UTR and exon 1 at 70,486,623–70,486,961 bp.
(TIF)

Figure S4 Expression pattern of *PDSS2* and *SOBP*. RT-PCR analysis of *PDSS2* and *SOBP* expression levels in multiple chicken tissues in (A) *H/H* and (B) *h/h* genotypes.
(TIF)

Figure S5 Spliced transcript analysis of *PDSS2* gene. (A) Schematic structure of the *PDSS2* gene and primers used for RT-PCR. (B) RT-PCR is performed with cDNA from dorsal skin and liver tissues of homozygous *H/H* and *h/h* individuals.
(TIF)

Figure S6 Spliced transcript analysis of *SOBP* gene. (A) Schematic structure of the *SOBP* gene and primers used for RT-PCR. (B) RT-PCR is performed with cDNA from dorsal skin and liver tissues that are from homozygous *H/H* and *h/h* individuals.
(TIF)

Figure S7 Relative mRNA expression levels of the other flanking genes around *silky-feather* mutation in skin. (A) The schematic structure of the flanking genes around *silky-feather* mutation *PDSS2(-103C-G)*. Relative mRNA expression of (B) *OSTM1*, (C) *SEC63*, (D) *SCML4*, (E) *BEND3*, (F) *C6orf203*, (G) *QRSL1*, (H) *RTN4IP1*, (I) *AIM1* and (J) *ENSGALT00000037260* gene in skin. Relative mRNA expression is compared with *GAPDH* gene. *H/H* and *h/h* represent *wild-type* homozygous and *silky-feather* homozygous birds separately. Dorsal skin is from postnatal (P) 10, 60, 130 and 200 birds. * indicates p<0.05 and *** indicates p<0.001. The bar represents standard deviation.
(TIF)

Figure S8 The chicken PDSS2 antibody is specific to the PDSS2 protein. (A) The chicken amino acid residues used for generating the anti-chicken PDSS2 antibody and its homologous sequence in human. (B) Western blotting analysis demonstrates that both cPDSS2 and GFP antibodies bind to the eGFP-cPDSS2 fusion protein (predicted molecular weight: 68.1 kDa) from the transfected 293T cells. The cPDSS2 antibody also binds to the endogenous PDSS2 protein from the DF1 (predicted molecular weight of cPDSS2: 41.2 kDa) and 293T cells (predicted molecular weight of human PDSS2: 44.1 kDa). 293T-WT, wild-type 293T cells; DF1-WT, wild-type DF1 cells; 293T-Trans, 293T cells transfected with pcDNA-eGFP-cPDSS2. (C–F) Immunofluorescence experiments in 293T and DF1 cells. The eGFP-cPDSS2 fusion protein (green) is fully consistent with cPDSS2 antibody pattern (red), as indicated by arrows in transfected GFP-positive 293T (C) and DF1 (D) cells. The cPDSS2 antibody also recognizes the endogenous cPDSS2 protein with weak signals (red), as indicated by arrowheads in GFP-negative DF1 (D, F) cells. Red staining indicates that PDSS2 protein is localized in the cytoplasm. Scale bar, 20 µm.
(TIF)

Table S1 SNP marker information used for mapping.
(PDF)

Table S2 Frequency analysis of *silky-feather* in different breeds. The adjacent SNPs, whose alleles are fixed in silky-feather birds, are highlighted in yellow.
(PDF)

Table S3 Exon SNP information in *SOBP* and *PDSS2* and genotype distribution. Four SNPs in the exons of *SOBP* and nine SNPs in the exons of *PDSS2* gene were identified, and genotyped in three different populations with known silky-feather genotypes. None of these SNP genotypes was completely associated with silky-feather phenotype, vice versa.
(PDF)

Table S4 Genotype results for ss666793770 of *PDSS2* in different populations. The *PDSS2* exon 5 G682A; Glu228Lys mutation (ss666793770) is predicted to cause a gain of MoRF binding (P = 0.026) and a gain of ubiquitination (P = 0.045) [80]. ss666793770 is genotyped in the larger population and confirmed to not associated with *silky-feather*. The A mutant allele is present in some heterozygous G/A birds with low frequency (freq A = 0.032) and the heterozygous birds show wild-type normal feather. Thus ss666793770 is excluded for further research.
(PDF)

Table S5 Primer sequences information.
(PDF)
