# Supplementary figures and images for: A cis-Regulatory Mutation of PDSS2 Causes Silky-Feather in Chickens
Source: PLoS Genet. 2014 Aug 28;10(8):e1004576. doi: 10.1371/journal.pgen.1004576 (PMC4148213; doi:10.1371/journal.pgen.1004576)

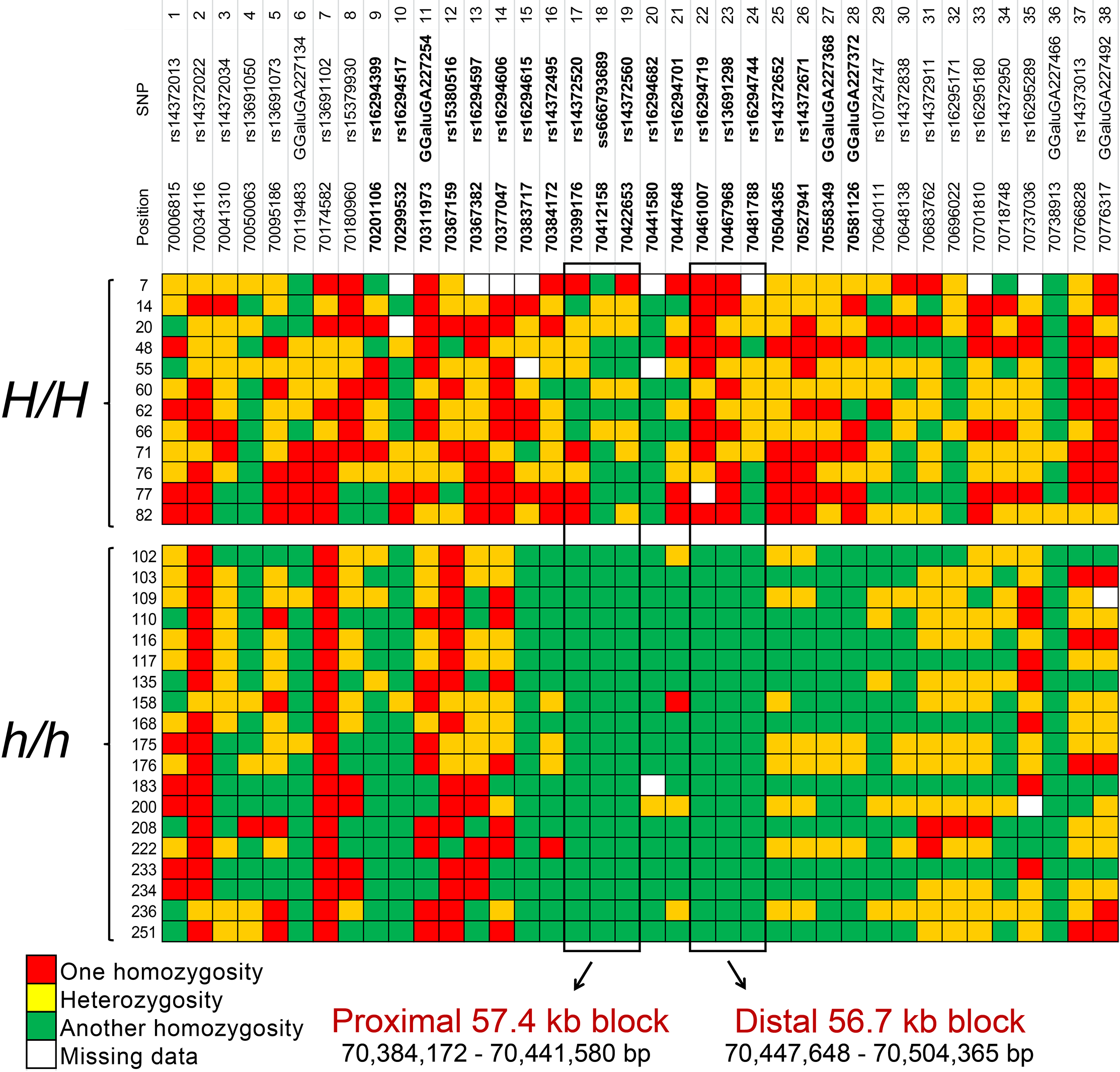

Supplement: Figure S1 — IBD mapping in CAURP narrows the silky-feather locus to 56.7 kb interval. The genotypes of 38 SNP markers covering about 770 kb interval (70,006,815–70,776,317 bp) in F0 individuals are shown. The silky-feather is mapped to the 380-kb interval (70,201,106–70,581,126 bp) by linkage analysis. H/H represents wild-type homozygotes and h/h represents silky-feather allele homozygotes. Red and Green: two alternative homozygous genotypes. Yellow: heterozygous genotype. White: missing genotype. Homozygosity for the Silkie birds is limited to two short haplotype blocks marked by black thick lines respectively: the proximal 57.4-kb (70,384,172–70,441,580 bp) and the distal 56.7-kb (70,447,648–70,504,365 bp). The distal block is exclusively shared by Silkie birds. (TIF) [file pgen.1004576.s001.tif]

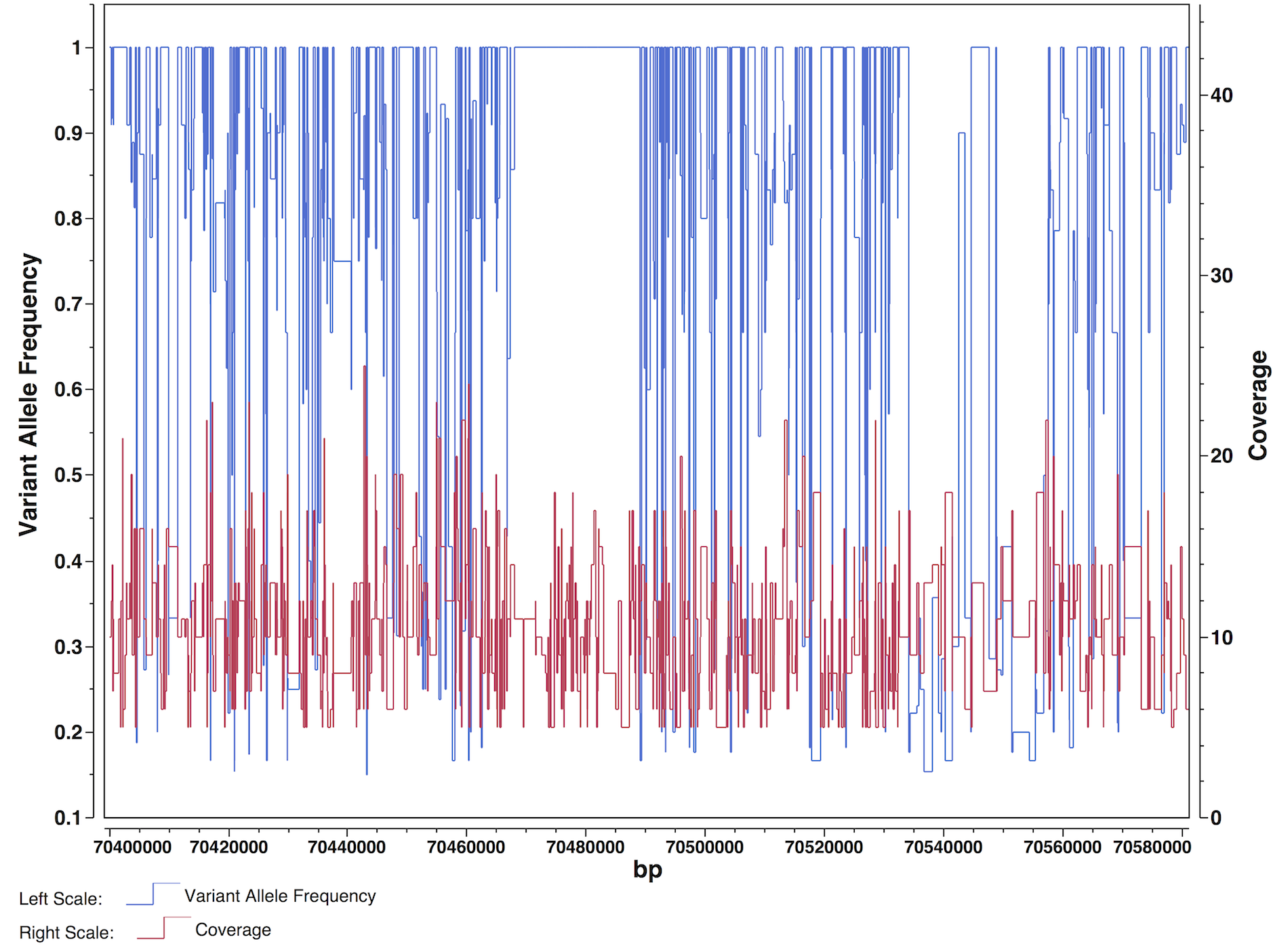

Supplement: Figure S2 — IBD haplotype detected by whole genome sequencing in Silkies from USA. A single extended genomic region fixed for the variant allele at all detected SNPs and representing an IBD haplotype is found in a pool of 15 Silkie chickens from the USA by whole genome sequencing. Variant allele frequency (left y-axis) is indicated in blue and coverage (right y-axis) is indicated in red. The IBD haplotype in the USA Silkies is 21.7 kb (70,467,293–70,489,020 bp) and completely overlaps the 18.9 kb IBD haplotype identified in Silkies from China. (TIF) [file pgen.1004576.s002.tif]

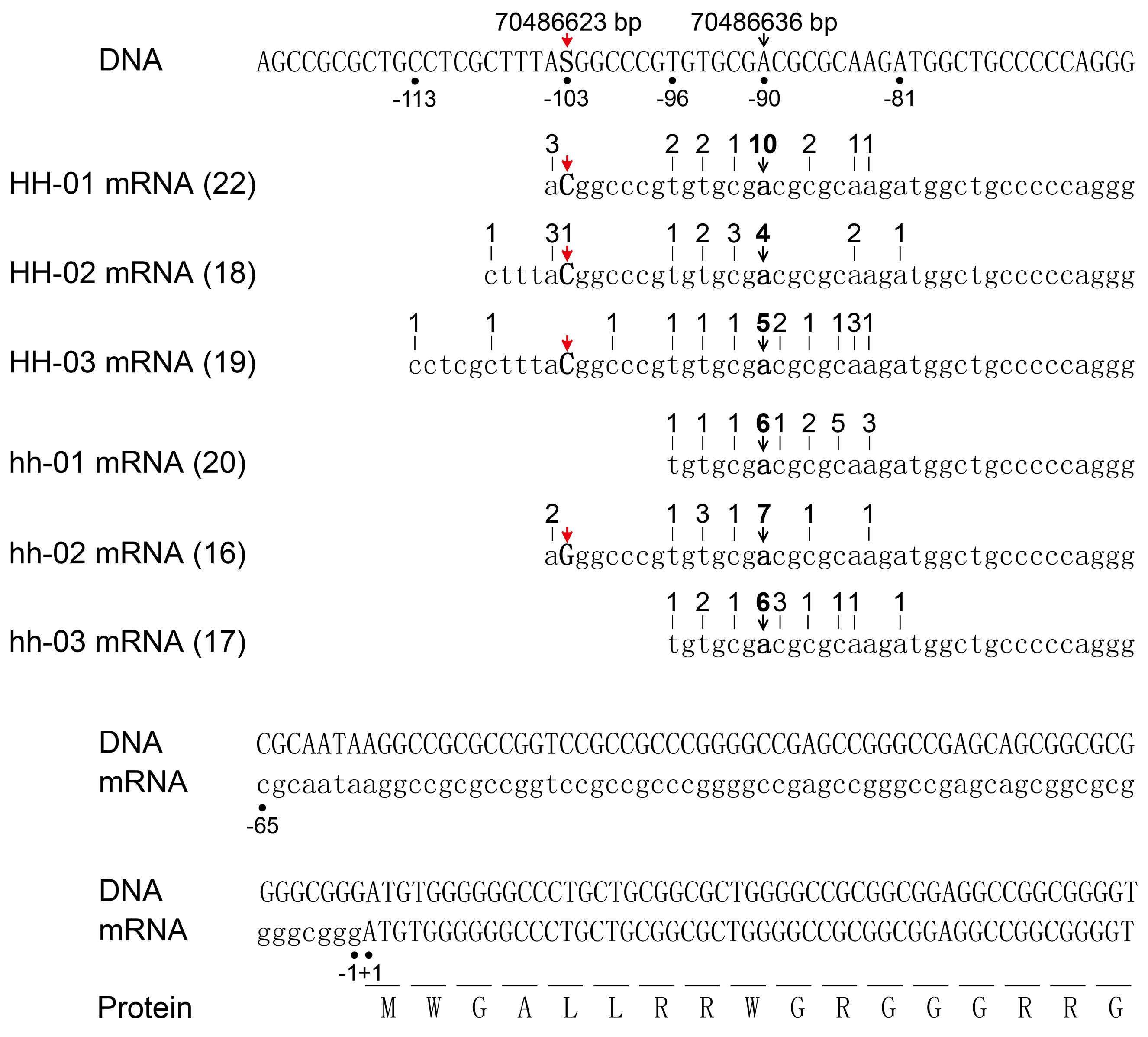

Supplement: Figure S3 — The 5′ RACE analysis of PDSS2 in skin tissue. Figure shows the DNA, mRNA and protein information of part of PDSS2 in skin. Three wild-type (H/H) and three silky-feather (h/h) dorsal skin tissues are used for 5′ RACE analysis as described in Materials and Methods. The total number of random RACE clones for each sample is included in the bracket. The 5′ ends of clones are indicated by the vertical lines with Arabic numerals to indicate the number of clones isolated for each site. The A of the translation start site (ATG) is defined as position +1. The nucleotide positions relative to the ATG are marked with solid circle under each site. The causative mutation PDSS2(-103C-G) (70,486,623 bp) is highlighted in uppercase and indicated by the red arrow. The most common start site (70,486,636 bp) is highlighted in bold and indicated by the black arrow. Most of the 5′ ends are located between position −81 and −96 around the most common position −90. Specifically, the proportions from all the six samples are 86.4% (19/22, HH-01), 72.2% (13/18, HH-02), 84.2% (16/19, HH-03), 100% (20/20, hh-01), 87.5% (14/16, hh-02) and 100% (17/17, hh-03), respectively. Furthermore, PDSS2(-103C-G) is contained in a few clones. Determining the exact transcription start site of PDSS2 with 5′ RACE is technically challenging due to the high GC content (75.8%) in 339-bp sequence of the presumed 5′ UTR and exon 1 at 70,486,623–70,486,961 bp. (TIF) [file pgen.1004576.s003.tif]

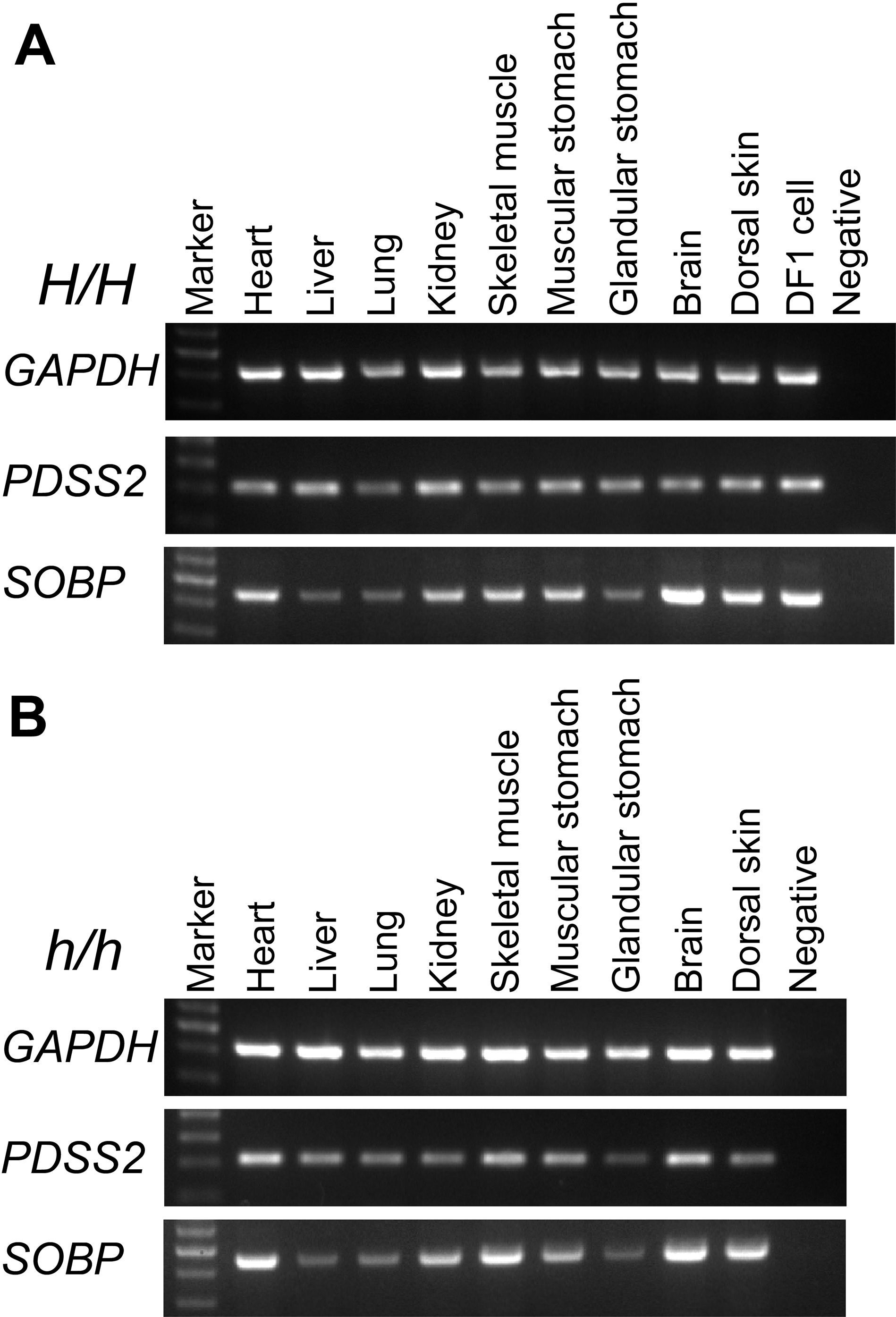

Supplement: Figure S4 — Expression pattern of PDSS2 and SOBP. RT-PCR analysis of PDSS2 and SOBP expression levels in multiple chicken tissues in (A) H/H and (B) h/h genotypes. (TIF) [file pgen.1004576.s004.tif]

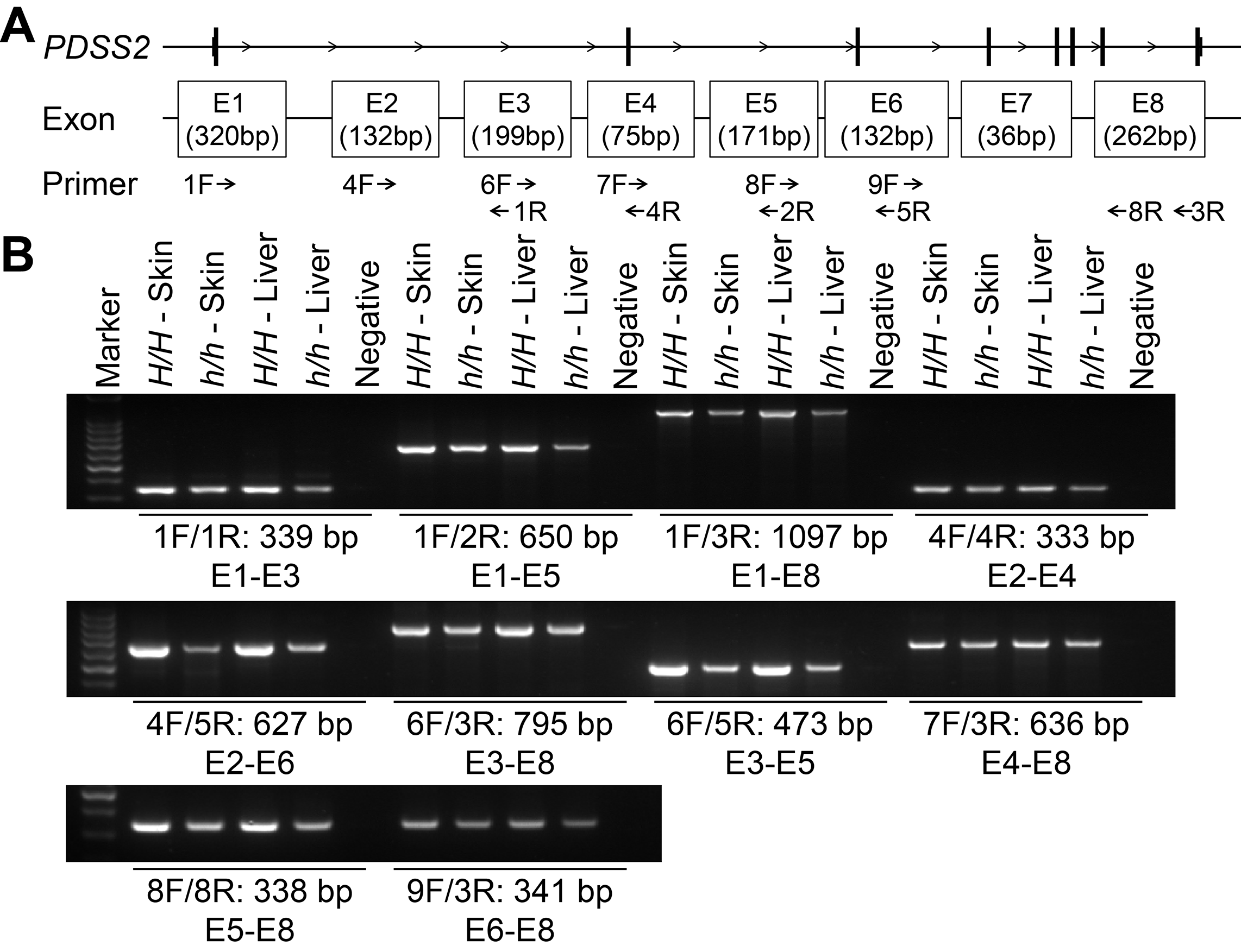

Supplement: Figure S5 — Spliced transcript analysis of PDSS2 gene. (A) Schematic structure of the PDSS2 gene and primers used for RT-PCR. (B) RT-PCR is performed with cDNA from dorsal skin and liver tissues of homozygous H/H and h/h individuals. (TIF) [file pgen.1004576.s005.tif]

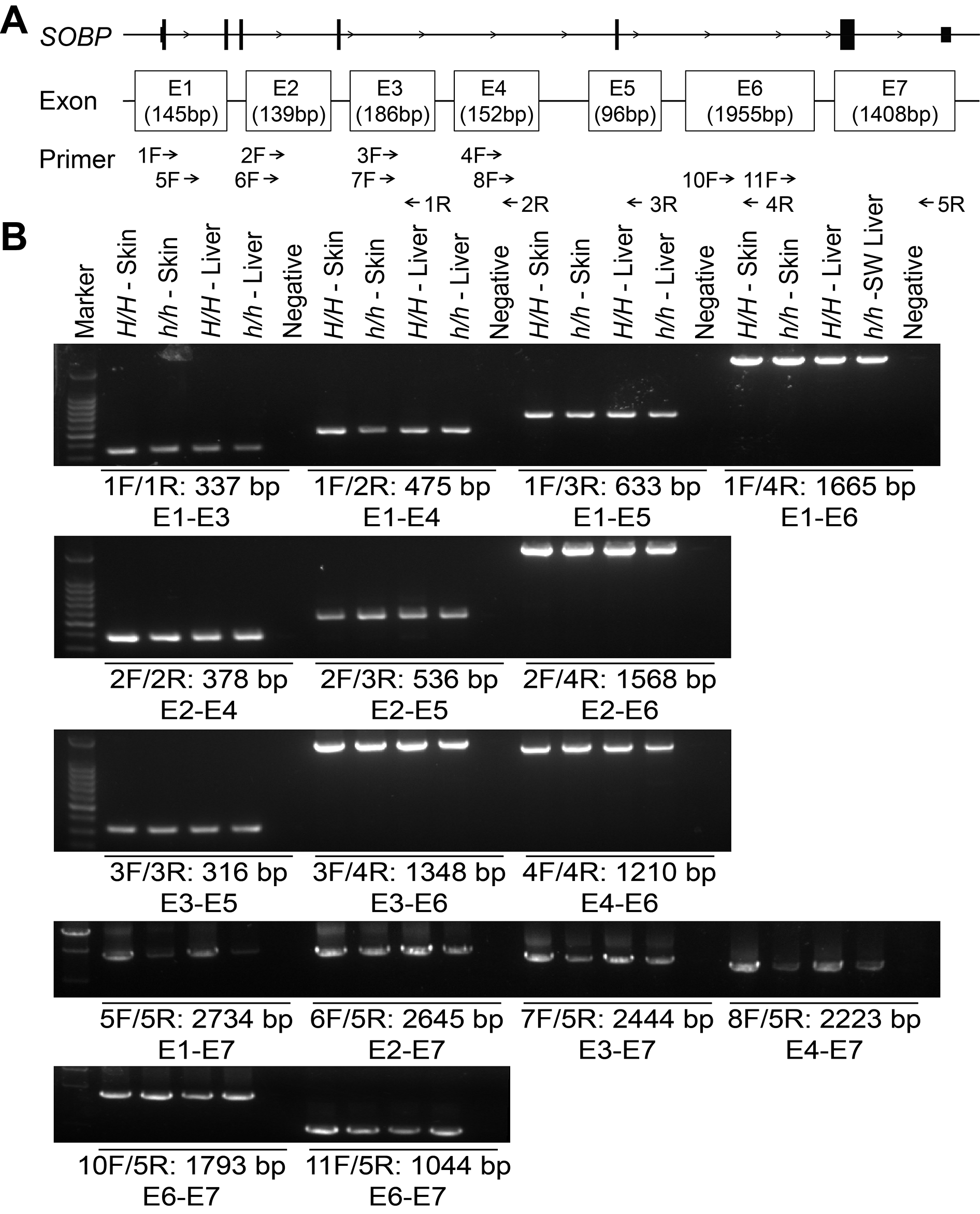

Supplement: Figure S6 — Spliced transcript analysis of SOBP gene. (A) Schematic structure of the SOBP gene and primers used for RT-PCR. (B) RT-PCR is performed with cDNA from dorsal skin and liver tissues that are from homozygous H/H and h/h individuals. (TIF) [file pgen.1004576.s006.tif]

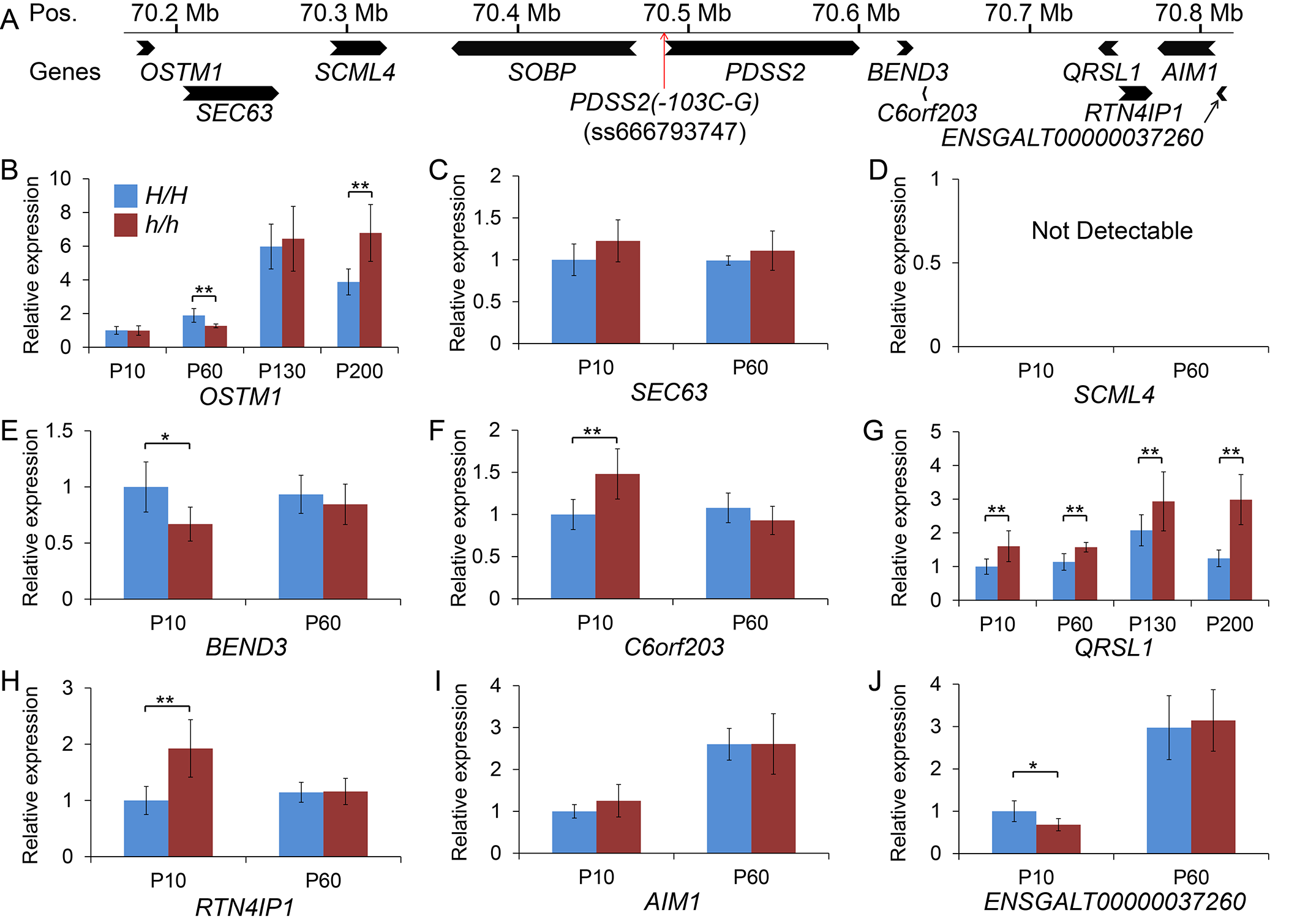

Supplement: Figure S7 — Relative mRNA expression levels of the other flanking genes around silky-feather mutation in skin. (A) The schematic structure of the flanking genes around silky-feather mutation PDSS2(-103C-G). Relative mRNA expression of (B) OSTM1, (C) SEC63, (D) SCML4, (E) BEND3, (F) C6orf203, (G) QRSL1, (H) RTN4IP1, (I) AIM1 and (J) ENSGALT00000037260 gene in skin. Relative mRNA expression is compared with GAPDH gene. H/H and h/h represent wild-type homozygous and silky-feather homozygous birds separately. Dorsal skin is from postnatal (P) 10, 60, 130 and 200 birds. * indicates p<0.05 and *** indicates p<0.001. The bar represents standard deviation. (TIF) [file pgen.1004576.s007.tif]

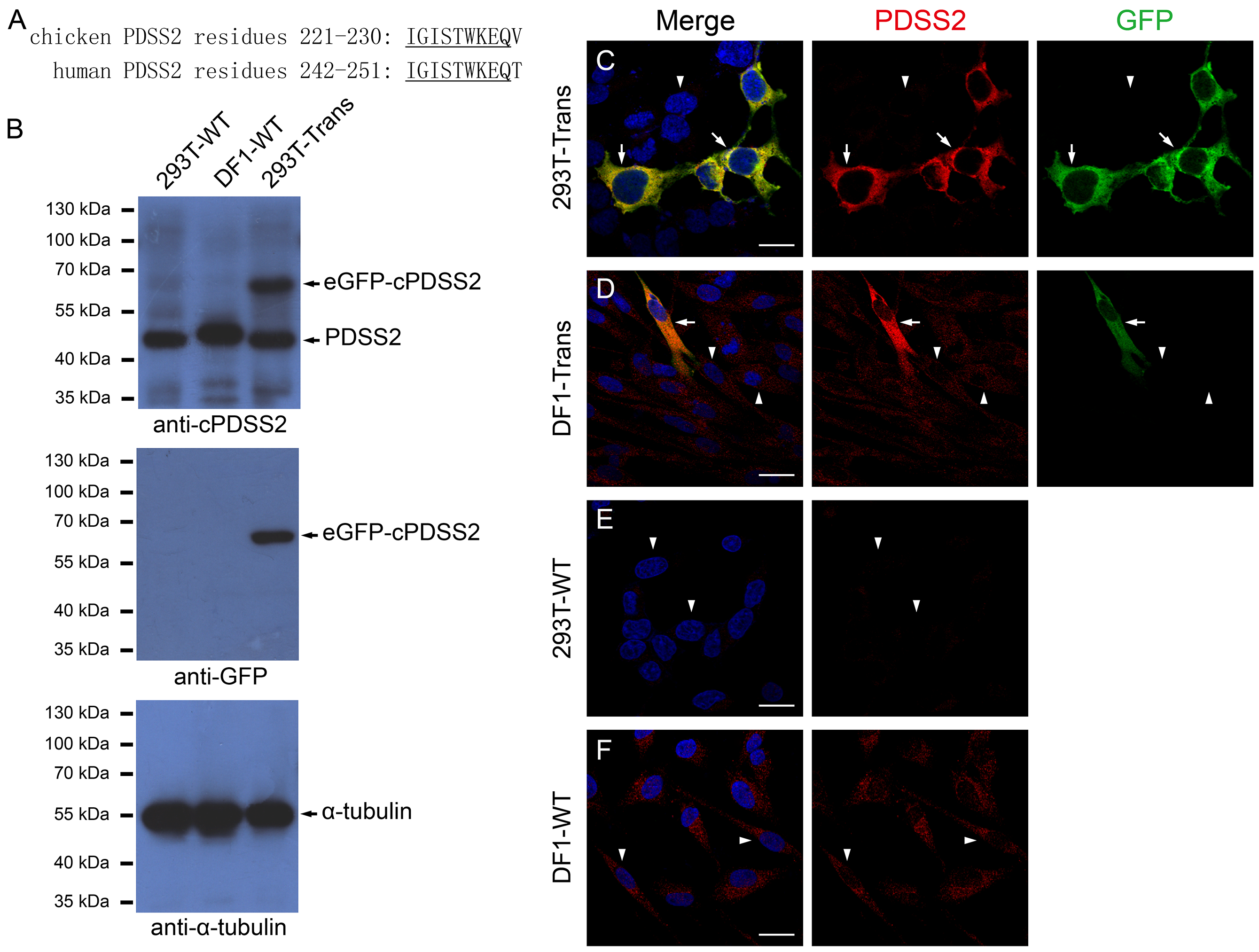

Supplement: Figure S8 — The chicken PDSS2 antibody is specific to the PDSS2 protein. (A) The chicken amino acid residues used for generating the anti-chicken PDSS2 antibody and its homologous sequence in human. (B) Western blotting analysis demonstrates that both cPDSS2 and GFP antibodies bind to the eGFP-cPDSS2 fusion protein (predicted molecular weight: 68.1 kDa) from the transfected 293T cells. The cPDSS2 antibody also binds to the endogenous PDSS2 protein from the DF1 (predicted molecular weight of cPDSS2: 41.2 kDa) and 293T cells (predicted molecular weight of human PDSS2: 44.1 kDa). 293T-WT, wild-type 293T cells; DF1-WT, wild-type DF1 cells; 293T-Trans, 293T cells transfected with pcDNA-eGFP-cPDSS2. (C–F) Immunofluorescence experiments in 293T and DF1 cells. The eGFP-cPDSS2 fusion protein (green) is fully consistent with cPDSS2 antibody pattern (red), as indicated by arrows in transfected GFP-positive 293T (C) and DF1 (D) cells. The cPDSS2 antibody also recognizes the endogenous cPDSS2 protein with weak signals (red), as indicated by arrowheads in GFP-negative DF1 (D, F) cells. Red staining indicates that PDSS2 protein is localized in the cytoplasm. Scale bar, 20 µm. (TIF) [file pgen.1004576.s008.tif]
