# Supplementary material for: A cis-Regulatory Mutation of PDSS2 Causes Silky-Feather in Chickens
Source: PLoS Genet. 2014 Aug 28;10(8):e1004576. doi: 10.1371/journal.pgen.1004576 (PMC4148213; doi:10.1371/journal.pgen.1004576)
Supplement: Table S1 — SNP marker information used for mapping. (PDF) [file pgen.1004576.s009.pdf]

Table S1. SNP marker information used for mapping

	SNP Marker	Position (bp)	Allele 1	Allele 2	Genotyping method	Purpose
1	ss666793685	69,861,956	A	G	SNPlex	Linkage
2	rs14372034	70,041,310	A	G	SNPlex	Linkage
3	rs16294479	70,264,459	T	A	SNPlex	Linkage
4	rs16294517	70,299,532	A	G	SNPlex	Linkage
5	rs16294597	70,367,382	T	C	SNPlex	Linkage
6	rs16294606	70,377,047	C	T	SNPlex	Linkage
7	rs16294615	70,383,717	C	A	SNPlex	Linkage
8	ss666793689	70,412,158	C	T	SNPlex	Linkage
9	rs16294682	70,441,580	T	A	SNPlex	Linkage
10	rs16294719	70,461,007	C	T	SNPlex	Linkage
11	rs13691298	70,467,968	A	G	SNPlex	Linkage
12	rs16294744	70,481,788	A	G	SNPlex	Linkage
13	rs16295180	70,701,810	T	C	SNPlex	Linkage
14	rs16295289	70,737,036	C	T	SNPlex	Linkage
15	rs14373412	71,162,959	C	T	SNPlex	Linkage
16	rs16296251	71,393,087	G	A	SNPlex	Linkage
17	rs16294682	70,441,580	T	A	Sequenom	IBD
18	rs16294685	70,443,091	T	A	Sequenom	IBD
19	ss189592165	70,447,516	G	A	Sequenom	IBD
20	rs16294701	70,447,648	A	G	Sequenom	IBD
21	rs14372603	70,447,861	C	T	Sequenom	IBD
22	rs13691273	70,448,626	G	A	Sequenom	IBD
23	rs13691274	70,448,826	T	G	Sequenom	IBD
24	rs15380763	70,449,233	G	A	Sequenom	IBD
25	ss189592233	70,450,972	C	T	Sequenom	IBD
26	ss189592329	70,453,700	T	C	Sequenom	IBD
27	rs16294708	70,454,995	A	G	Sequenom	IBD
28	ss189592368	70,455,598	A	G	Sequenom	IBD
29	rs15380817	70,458,173	C	T	Sequenom	IBD
30	ss189592514	70,459,811	A	G	Sequenom	IBD
31	rs13691287	70,460,350	G	A	Sequenom	IBD
32	ss666793690	70,460,490	G	C	Sequenom	IBD
33	ss189592554	70,460,738	T	C	Sequenom	IBD
34	rs16294720	70,461,033	G	C	Sequenom	IBD
35	ss189592667	70,464,192	A	G	Sequenom	IBD
36	ss189592682	70,464,722	T	C	Sequenom	IBD
37	ss189592760	70,466,320	T	C	Sequenom	IBD
38	ss666793713	70,466,750	A	T	Sequenom	IBD
39	rs13691298	70,467,968	A	G	Sequenom	IBD
40	ss189592835	70,471,618	T	C	Sequenom	IBD
41	ss666793721	70,472,921	C	T	Sequenom	IBD

42	ss189592886	70,473,176	G	A	Sequenom	IBD
43	ss189592940	70,474,481	T	C	Sequenom	IBD
44	ss189592951	70,474,838	C	T	Sequenom	IBD
45	ss189593025	70,476,291	G	A	Sequenom	IBD
46	ss189593037	70,476,573	C	T	Sequenom	IBD
47	rs15380871	70,476,827	G	A	Sequenom	IBD
48	ss666793742	70,479,823	T	A	Sequenom	IBD
49	rs16294744	70,481,788	A	G	Sequenom	IBD
50	ss189593326	70,484,959	A	G	Sequenom	IBD
