# Supplementary material for: A cis-Regulatory Mutation of PDSS2 Causes Silky-Feather in Chickens
Source: PLoS Genet. 2014 Aug 28;10(8):e1004576. doi: 10.1371/journal.pgen.1004576 (PMC4148213; doi:10.1371/journal.pgen.1004576)
Supplement: Table S2 — Frequency analysis of silky-feather in different breeds. The adjacent SNPs, whose alleles are fixed in silky-feather birds, are highlighted in yellow. (PDF) [file pgen.1004576.s010.pdf]

Table S2. Frequency analysis of *silky-feather* in different breeds

The adjacent SNPs, whose alleles are fixed in silky-feather birds, are highlighted in yellow.

Phenotype			**Silky-feather**			**Wild-type**								
Breed			Silkie	Lanping Silky	Kuaida Silky	White Plymouth Rock	Red Jungle Fowl	Chahua	Langshan	Tibetan	White Leghorn	Anak	Recessive White Rock	Henan Game
Sample Size			50	16	10	8	20	10	10	10	10	10	10	7
SNP	Position (bp)	Allele												
rs16294682	70,441,580	T	0.84	0.78	0.70	1.00	0.53	0.65	0.10	0.83	0.90	0.90	0.55	0.64
rs16294685	70,443,091	T	1.00	1.00	1.00	1.00	0.85	0.80	1.00	1.00	1.00	1.00	1.00	1.00
ss189592165	70,447,516	G	0.83	1.00	0.67	0.88	0.95	0.40	1.00	0.20	0.00	0.79	0.79	1.00
rs16294701	70,447,648	G	0.75	0.81	0.60	0.50	0.37	0.35	0.20	0.15	0.35	0.35	0.25	0.83
rs14372603	70,447,861	C	0.76	1.00	0.60	0.50	0.75	0.45	0.75	0.30	0.00	0.65	0.60	0.83
rs13691273	70,448,626	A	1.00	1.00	1.00	0.94	0.95	0.95	0.85	0.83	0.85	1.00	0.90	0.57
rs13691274	70,448,826	G	1.00	0.81	1.00	0.71	0.70	0.90	0.72	0.72	0.35	0.39	0.78	0.21
rs15380763	70,449,233	A	1.00	1.00	1.00	0.93	0.85	1.00	1.00	1.00	0.60	1.00	1.00	1.00
ss189592233	70,450,972	T	0.80	0.80	0.65	0.00	0.13	0.20	0.00	0.00	0.44	0.25	0.00	0.00
ss189592329	70,453,700	T	1.00	1.00	1.00	1.00	0.83	1.00	1.00	1.00	1.00	1.00	1.00	0.86
rs16294708	70,454,995	G	0.80	0.75	0.65	0.07	0.55	0.20	0.20	0.10	0.80	0.25	0.06	0.90
ss189592368	70,455,598	G	0.80	0.94	0.65	0.50	0.45	0.45	0.20	0.35	1.00	0.90	0.35	0.75
rs15380817	70,458,173	T	1.00	1.00	1.00	0.67	0.60	1.00	0.60	0.86	1.00	0.60	1.00	0.67
ss189592514	70,459,811	G	0.84	0.94	0.70	0.75	0.73	0.55	0.70	0.56	1.00	1.00	0.65	0.57
rs13691287	70,460,350	A	1.00	1.00	1.00	1.00	0.88	1.00	0.44	0.89	0.94	1.00	1.00	1.00
ss666793690	70,460,490	G	0.83	0.94	0.70	0.06	0.40	0.30	0.25	0.28	0.25	0.00	0.10	0.00
ss189592554	70,460,738	C	0.98	0.94	1.00	0.00	0.05	0.25	0.20	0.25	0.25	0.00	0.06	0.00
**rs16294720**	**70,461,033**	**G**	**1.00**	**1.00**	**1.00**	0.25	0.64	0.80	1.00	0.94	0.75	0.00	0.57	0.93
**ss189592667**	**70,464,192**	**G**	**1.00**	**1.00**	**1.00**	0.88	0.95	1.00	1.00	1.00	0.35	0.70	0.90	1.00
**ss189592682**	**70,464,722**	**C**	**1.00**	**1.00**	**1.00**	0.63	0.38	0.65	1.00	0.67	0.65	0.55	0.45	0.43

**ss189592760**	**70,466,320**	**C**	**1.00**	**1.00**	**1.00**	1.00	0.98	0.90	1.00	0.83	1.00	1.00	1.00	0.86
**ss666793713**	**70,466,750**	**A**	**0.98**	**1.00**	**1.00**	0.20	0.03	0.05	0.15	0.15	0.90	0.35	0.17	0.00
**rs13691298**	**70,467,968**	**G**	**1.00**	**1.00**	**1.00**	0.36	0.43	0.25	0.45	0.40	0.70	0.30	0.25	0.83
**ss189592835**	**70,471,618**	**C**	**1.00**	**1.00**	**1.00**	0.00	0.30	0.05	0.50	0.25	0.45	0.22	0.20	0.21
**ss666793721**	**70,472,921**	**T**	**1.00**	**1.00**	**1.00**	0.50	0.30	0.80	0.65	0.61	0.60	0.15	0.45	0.07
**ss189592886**	**70,473,176**	**A**	**1.00**	**1.00**	**1.00**	0.93	0.68	0.90	0.80	0.75	0.60	1.00	0.80	0.21
**ss189592940**	**70,474,481**	**C**	**1.00**	**1.00**	**1.00**	1.00	0.95	0.65	1.00	0.94	1.00	1.00	1.00	1.00
**ss189592951**	**70,474,838**	**T**	**1.00**	**1.00**	**1.00**	0.31	0.18	0.00	0.05	0.00	0.30	0.15	0.10	0.00
**ss189593025**	**70,476,291**	**A**	**1.00**	**1.00**	**1.00**	0.38	0.13	0.20	0.20	0.17	0.30	0.15	0.10	0.00
**ss189593037**	**70,476,573**	**T**	**1.00**	**1.00**	**1.00**	0.31	0.13	0.00	0.05	0.00	0.30	0.15	0.10	0.00
**rs15380871**	**70,476,827**	**A**	**1.00**	**1.00**	**1.00**	0.64	0.73	0.50	0.80	0.75	0.35	0.40	0.65	0.67
**ss666793742**	**70,479,823**	**T**	**1.00**	**1.00**	**1.00**	0.50	0.53	0.15	0.56	0.28	0.28	0.31	0.56	0.50
**rs16294744**	**70,481,788**	**G**	**1.00**	**1.00**	**1.00**	0.71	0.48	0.60	1.00	0.45	0.90	0.45	0.65	0.42
**ss189593326**	**70,484,959**	**G**	**1.00**	**1.00**	**1.00**	0.70	0.21	0.00	0.55	0.25	0.28	0.44	0.55	0.30

,
