# Supplementary material for: A cis-Regulatory Mutation of PDSS2 Causes Silky-Feather in Chickens
Source: PLoS Genet. 2014 Aug 28;10(8):e1004576. doi: 10.1371/journal.pgen.1004576 (PMC4148213; doi:10.1371/journal.pgen.1004576)
Supplement: Table S3 — Exon SNP information in SOBP and PDSS2 and genotype distribution. Four SNPs in the exons of SOBP and nine SNPs in the exons of PDSS2 gene were identified, and genotyped in three different populations with known silky-feather genotypes. None of these SNP genotypes was completely associated with silky-feather phenotype, vice versa. (PDF) [file pgen.1004576.s011.pdf]

Table S3. Exon SNP information in *SOBP* and *PDSS2* and genotype distribution

Four SNPs in the exons of *SOBP* and nine SNPs in the exons of *PDSS2* gene were identified, and genotyped in three different populations with known *silky-feather* genotypes. None of these SNP genotypes was completely associated with *silky-feather* phenotype, vice versa.

SNP ID	Position (bp)	Gene	Exon	Reference Allele	Alternative Allele	Genotype[a]	Wild homozygote (HH)[b]			Heterozygote (Hh)[b]			Mutant homozygote (hh)[b]		
							Ref/Ref	Ref/Alt	Alt/Alt	Ref/Ref	Ref/Alt	Alt/Alt	Ref/Ref	Ref/Alt	Alt/Alt
ss666793686	70,373,762	*SOBP*	6	C	T		6	1	0	0	12	3	1	0	2
ss189590000	70,375,083	*SOBP*	6	T	C		3	3	1	0	3	11	1	0	2
ss666793687	70,375,194	*SOBP*	6	G	A		6	1	0	3	12	0	1	0	2
ss666793688	70,375,212	*SOBP*	6	G	A		6	1	0	3	12	0	1	0	2
ss666793766	70,534,744	*PDSS2*	2	T	C		7	0	0	12	3	0	3	0	0
ss189595641	70,561,353	*PDSS2*	3	G	A		5	0	2	12	2	1	3	0	0
ss189595959	70,576,500	*PDSS2*	4	C	T		1	1	5	4	7	4	3	0	0
ss666793770	70,584,425	*PDSS2*	5	G	A		6	1	0	11	4	0	3	0	0
ss666793771	70,584,426	*PDSS2*	5	G	A		6	1	0	11	4	0	3	0	0
ss666793772	70,584,500	*PDSS2*	5	C	T		6	1	0	15	0	0	3	0	0
ss189596174	70,586,288	*PDSS2*	6	C	T		6	1	0	2	9	4	0	0	3
ss189596728	70,600,783	*PDSS2*	8	C	T		0	6	1	12	3	0	3	0	0
ss666793773	70,600,816	*PDSS2*	8	T	C		1	6	0	1	11	3	3	0	0

[a]Ref stands for the Reference Allele and Alt stands for the Alternative Allele.

[b]Wild homozygote contains seven Youxi Partridge birds; heterozygote contains 15 birds from a cross between Youxi Partridge and Silkie chicken; mutant homozygote contains three Silkie birds.
