# Supplementary material for: A cis-Regulatory Mutation of PDSS2 Causes Silky-Feather in Chickens
Source: PLoS Genet. 2014 Aug 28;10(8):e1004576. doi: 10.1371/journal.pgen.1004576 (PMC4148213; doi:10.1371/journal.pgen.1004576)
Supplement: Table S4 — Genotype results for ss666793770 of PDSS2 in different populations. The PDSS2 exon 5 G682A; Glu228Lys mutation (ss666793770) is predicted to cause a gain of MoRF binding (P = 0.026) and a gain of ubiquitination (P = 0.045) [80]. ss666793770 is genotyped in the larger population and confirmed to not associated with silky-feather. The A mutant allele is present in some heterozygous G/A birds with low frequency (freq A = 0.032) and the heterozygous birds show wild-type normal feather. Thus ss666793770 is excluded for further research. (PDF) [file pgen.1004576.s012.pdf]

Table S4. Genotype results for ss666793770 of *PDSS2* in different populations

The *PDSS2* exon 5 G682A; Glu228Lys mutation (ss666793770) was predicted to cause a gain of MoRF binding (P = 0.026) and a gain of ubiquitination (P = 0.045) [64]. ss666793770 was genotyped in the larger population and confirmed to not associated with *silky-feather*. The A mutant allele was just present in some heterozygous G/A birds with low frequency (freq A = 0.032) and the heterozygous birds showed wild-type normal feather. Thus ss666793770 was excluded for further research.

Breed	Genotype		
	G/G	A/G	A/A
**Silky-feather**			
Kuaida Silky	10	0	0
Silkie	51	0	0
**Heterozygote**			
Silkie crossbred	7	4	0
**Wild-type**			
Beijing You	6	0	0
Huiyang Bearded	8	0	0
Red Jungle Fowl	20	0	0
Youxi Partridge	73	8	0
**Total**	175	12	0
