# Supplementary material for: A cis-Regulatory Mutation of PDSS2 Causes Silky-Feather in Chickens
Source: PLoS Genet. 2014 Aug 28;10(8):e1004576. doi: 10.1371/journal.pgen.1004576 (PMC4148213; doi:10.1371/journal.pgen.1004576)
Supplement: Table S5 — Primer sequences information. (PDF) [file pgen.1004576.s013.pdf]

Table S5. Primer sequences information

A. Primer information used for microsatellite analysis

Primer	Sequence	Length (bp)	Position
SF01F	FAM-TGACCTTCTGCTCAGCGTTA	232	chr3:69276469+69276700
SF01R	TTTGGAAACGGTTGTTTCATC		
SF02F	HEX-TTAGCTCCCACAAGCCATTC	212	chr3:69564297+69564508
SF02R	GGAGAAGGAATAGGGATCAAGTC		
SF03F	FAM-ATTGGCAAGCAATTCAAGAT	161	chr3:70406403+70406563
SF03R	TGAAAACCGTTTCTCTTTCTG		
SF04F	FAM-AAATGGTGCAGTCCACAACA	264	chr3:70874924+70875187
SF04R	GCTGCACCTAGTTGGCATTA		
SF05F	FAM-AGAGGTGCAGATTCCATTCC	212	chr3:71413274+71413485
SF05R	CTTACAAAGTCCCGCTTGCT		
SF06F	FAM-CCAACCTTCCCTGGTCAGTA	210	chr3:71627748+71627957
SF06R	TACTGGAGGCCTGAAAATCG		
SF07F	HEX-ACGTCCTTTTCTTTGCAGAT	249	chr3:72032460+72032708
SF07R	CCAGTCCAGATGGTCTAAGG		
SF08F	FAM-GCCCAGGACATTGTCTAAG	238	chr3:72406805+72407042
SF08R	TTTCGGTGTCAGGATAGGAG		

B. Primer information used for resequencing

Primer	Sequence	Length (bp)	Position
Sil-seq-1F	TGAAGGTGGCTTAACAACAGTG	1111	chr3:70460253+70461363
Sil-seq-1R	AATGAGCTCCTTGGCTGGTA		
Sil-seq-2F	GCCAATTAGGCAGGTCAAGA	1135	chr3:70461180+70462314
Sil-seq-2R	GACCTCTGCAGTGACAACGA		
Sil-seq-3F	GGGCCAGGAGATTTAGTGGAGTAAG	910	chr3:70462166+70463075
Sil-seq-3R	TGAGGCATAATGGAGTGTCATAGGC		
Sil-seq-4F	CTTTTTCCCCTTGACCTCCT	1068	chr3:70462938+70464005
Sil-seq-4R	TAACAGTGCGGTGGGTTTTT		
Sil-seq-5F	CAGGGTCTTTGCAATTAACTTCAGC	938	chr3:70463866+70464803
Sil-seq-5R	CCACACTTGTCCTCACTTCACATTG		
Sil-seq-6F	GGAGCCAGCTGAACTGAGAGATG	989	chr3:70464733+70465721
Sil-seq-6R	CGCACTTCCTCCAAGATCTGAAA		
Sil-seq-7F	CTGCGCTTCTCTTCTCACCT	785	chr3:70465526+70466310
Sil-seq-7R	TATTCCCCGTTTGGCATTTA		

Sil-seq-8F	CTCTTTAACGTTTGCAGCTCTCTGG	978	chr3:70466201+70467178
Sil-seq-8R	TGCCTTGTGCCTGAGATTGAGTTAC		
Sil-seq-9F	AAAACGGTTCCCCTGAACAT	797	chr3:70467012+70467808
Sil-seq-9R	CTGGCTGTTTCCCTTCATCT		
Sil-seq-10F	TTTCTTTCTGCTCTGCCGTGATT	801	chr3:70467722+70468522
Sil-seq-10R	GTGCCCCTTCCTGCACAGTC		
Sil-seq-11F	TTCCTCACCCTTTCAGACAAACATC	1091	chr3:70468407+70469497
Sil-seq-11R	CTCCTTCCTCCCCTCTCTGCTC		
Sil-seq-12F	AGCCACCCCAACATCTGGAG	446	chr3:70469365+70469810
Sil-seq-12R	AGGCTGCAAATCCTGTTCATTGAC		
Sil-seq-13F	AAGAGCCCAACAGGGGAGAGTTTTT	338	chr3:70469738+70470075
Sil-seq-13R	AAATGGCAGAAATGGAGAAAGAAGG		
Sil-seq-14F	GAGGCGGAGTTGAGACACATTCAGT	1308	chr3:70469838+70471145
Sil-seq-14R	GGCTGTTTTGCGAAGTGTAAACGAG		
Sil-seq-15F	GGCTGAGTCCGACAGAAATC	851	chr3:70471077+70471927
Sil-seq-15R	TCAGCACTACGGAGAAACCA		
Sil-seq-16F	CCAACCTCACAGAACCTTCC	1011	chr3:70471802+70472812
Sil-seq-16R	CGAGTGTCAGATGGTGCCTA		
Sil-seq-17F	TTCCAGTGGCAACATATGGA	998	chr3:70472705+70473702
Sil-seq-17R	AGCCTCACAGGTGTCTTCGT		
Sil-seq-18F	TTCCAAAGCTGGGAAGAAAA	532	chr3:70473633+70474164
Sil-seq-18R	ACTGGAAAACTGGGCCTTCT		
Sil-seq-19F	TCTGGTCTTCATTAACGCGTAGCTC	503	chr3:70474058+70474560
Sil-seq-19R	CCCCGACTTTCAATTTCTGCTTATC		
Sil-seq-20F	TCCCTCCGTGGATAAGTCAG	793	chr3:70474368+70475160
Sil-seq-20R	CCTCCAAACATTTCCAGACC		
Sil-seq-21F	ATACACAGCCTCTCCGAAGC	657	chr3:70475069+70475725
Sil-seq-21R	GATGTGTGCCTCTCCCTGAT		
Sil-seq-22F	TTTGACTCTGGGGAAAATGC	1104	chr3:70475594+70476697
Sil-seq-22R	GGGGAATTTGGACAAAATGA		
Sil-seq-23F	ACGTCTGTCAAGGGATGGAA	1244	chr3:70476571+70477814
Sil-seq-23R	TCTCCCGGAGTTCAACAAAG		
Sil-seq-24F	CGCCTTGTTGGCTAATGTTC	1112	chr3:70477663+70478774
Sil-seq-24R	GGATGGATGTGAGGCTGACT		
Sil-seq-25F	CATGGAGAATGGGAGAGGTG	1266	chr3:70478670+70479935
Sil-seq-25R	AGTCAGCCTCGCAGTGAAAT		
Sil-seq-26F	GGACTGCTTACCTCAAATCACTG	1216	chr3:70479857+70481072

Sil-seq-26R	GCCTCTTCAATACGTGCTCTAAA		
Sil-seq-27F	GACCTTGTTCCCGGAAAGACATTAC	912	chr3:70481481+70482392
Sil-seq-27R	AGAGTAAATGACAGCCGTGCTGAAC		
Sil-seq-28F	CACTGCATGTTGTCTCCTACTCA	1205	chr3:70482134+70483338
Sil-seq-28R	AGCCTCACGCTCTGGATAGA		
Sil-seq-29F	TCATTCATTAACCAGCAACAAATC	1254	chr3:70483228+70484481
Sil-seq-29R	AATGATAGACAACCCTACCCAGAG		
Sil-seq-30F	GGTGCATCACACTTTTGTTTCT	1238	chr3:70484350+70485587
Sil-seq-30R	CGAGCCCTGTCTATTCTTGC		
Sil-seq-31F	ATTCTCCTTGCTCTGTTTGTACG	676	chr3:70485477+70486152
Sil-seq-31R	ACCATTCCATCACGAAACTAGAA		
Sil-seq-32F	CAGAGTGCACAGAAAGCAGACTTTG	662	chr3:70485834+70486495
Sil-seq-32R	GTCATACCCCTTGCTCCACACAG		
Sil-seq-33F	AGACGTGGCTGTTAGGAAGAGGTTC	752	chr3:70486339+70487090
Sil-seq-33R	GTCCCACTGTCTCCTCACAACAGG		
Sil-seq-34F	GGTGTCGGAAGCCGAGAAGA	1155	chr3:70486821+70487975
Sil-seq-34R	GTGCCTGACTGCGTTCCTGA		
Sil-seq-35F	AGCTGCTGGTTTGTTTGTGGATTAG	1022	chr3:70487807+70488828
Sil-seq-35R	CCAACCCAAACTGTTCTGTGATTCT		
Sil-seq-36F	AAAGCTTTTGAATGTGGTGCTGTTC	926	chr3:70488478+70489403
Sil-seq-36R	TGTCCTGTCTCACGCGGACTATTAT		
Sil-seq-37F	GTAAGTGGAGTGCTGATGAGCGTTT	910	chr3:70489271+70490180
Sil-seq-37R	CTGTGGCTCAGAAGGCTACAAAGAA		
Sil-seq-38F	GATCTTTCAGTGGAAAATGGTGTA	1166	chr3:70490027+70491192
Sil-seq-38R	CCTTTGTTTTAATAGCCCAGAAAA		
Sil-seq-39F	CATAATTGCGTGTGCTGTGA	1180	chr3:70491092+70492271
Sil-seq-39R	CCCCTTAACTGGGTGAGACA		
Sil-seq-40F	GGTTCACACGCAGATTGAAA	1237	chr3:70492153+70493389
Sil-seq-40R	CCACACACTGGAAACCCAAT		
Sil-seq-41F	TTTCTGCATGTCACACTCATCCATT	982	chr3:70492282+70493263
Sil-seq-41R	CAAGGCAGACACTCCTACAGACACA		
Sil-seq-42F	GAGTGTCTGCCTTGTGCTGA	1153	chr3:70493250+70494402
Sil-seq-42R	TGTCTCCTGCGGTAGTTCCT		
Sil-seq-43F	GTGGAAGGCCATTGTTTTCA	1305	chr3:70494279+70495583
Sil-seq-43R	AGGATGGCTTTACACCAACG		
Sil-seq-44F	CTCTCACTGGTCCATGTGTTGT	1201	chr3:70495450+70496650
Sil-seq-44R	GTCCCGTTCCTTGAACAAAT		

Sil-seq-45F	GAACTCAAGGAAGCTGGAAGG	1209	chr3:70496579+70497787
Sil-seq-45R	CGGTAATCCAAAGCAGTGGT		
Sil-seq-46F	CCCCGCTCTTATGCTGTAAA	1220	chr3:70497688+70498907
Sil-seq-46R	TGCATCGACAAATGATGACA		
Sil-seq-47F	ATCCTGCAGGCTGGTTATTG	1264	chr3:70498782+70500045
Sil-seq-47R	CAGGCATGGCAGTACCATATT		
Sil-seq-48F	ACCTTAAAGCCCGTTCGGTTC	1145	chr3:70500662+70501806
Sil-seq-48R	CTCTAGCCCAGGTTGCTCACA		
Sil-seq-49F	CATGGTAGTGAGACAGTGGAACAGG	1180	chr3:70501699+70502878
Sil-seq-49R	AGGTACACACCGAGGCTGAAAAAG		
Sil-seq-50F	GACTAACAGCTCCCCTCGTG	1206	chr3:70502767+70503972
Sil-seq-50R	CATCACTTAGCAGCCAAGCA		
Sil-seq-51F	TGGTAACCGTTCAGGGAGAA	1126	chr3:70503832+70504957
Sil-seq-51R	CAGGCATGACCTTGGCTATC		
Sil-seq-52F	TCCAGCCTTCTGAGGTGAGT	1136	chr3:70504837+70505972
Sil-seq-52R	GAAGAGGCCACCTGCTATTG		
Sil-seq-53F	TTTTCAGTGTGTGGGTTTGGTAGGT	1205	chr3:70505875+70507079
Sil-seq-53R	TGACCACATCGTTACATGGTCTGAT		
Sil-seq-54F	GCAGTGCCTCAGGATTACAGACATT	1024	chr3:70506894+70507917
Sil-seq-54R	AGTGAGGGACTGAAGTGCTCCTTTT		
Sil-seq-55F	CCGTCAAGGCATCACTAACA	1231	chr3:70507793+70509023
Sil-seq-55R	ATCGACTTTGGCATCTGCTT		
Sil-seq-56F	AGGGCACTCATCAAGGACAG	1228	chr3:70515823+70517050
Sil-seq-56R	TGGATTCCAGTTCCAAAGGA		
Sil-seq-57F	GCAATGATCCAGGAAAAGGA	1273	chr3:70525804+70527076
Sil-seq-57R	CGCCAACCAGTATCAACAAG		
Sil-seq-58F	CTTGCTAAGGAGGGGCTGT	1201	chr3:70535884+70537084
Sil-seq-58R	TGGCATGGTAACAGAAAGAGC		
Sil-seq-59F	TAGGCCCTGTTTGTGGACTC	1235	chr3:70545836+70547070
Sil-seq-59R	TACAACCAGCAAATGCCATC		

C. Primer information used for pyrosequencing

Primer	Sequence	Length (bp)	Purpose
70486623pyF	Biotin-CGACTCTCAACGCGGGAAC	123	ss666793747
70486623pyR	CTGGGGGCAGCCATCTTG		
70486623pyS	GCGTCGCACACGGGC		
70584425pyF	AGCTGTCACAGTTGCAGTTTTG	134	ss666793770

70584425pyR	Biotin-TCTTTGCCAGCAAGGCACTA		
70584425pyS	CATTGGAATATCTACGTG		
70373762pyF	Biotin-ACCTCAACAATCCGGCAGA	125	ss666793686
70373762pyR	TGACATAATACACGGTGCGATAG		
70373762pyS	CATACGCATGGTCCTC		
70375194pyF	CTGGCCAAATTCAAGGTCC	94	ss666793687
70375194pyR	Biotin-ACTGGCGTGGGGATTTTAGT		
70375194pyS	CGTCTACCACTGCCTC		
70586288pyF	TTCAGACCTTCAGCCATTTG	114	ss189596174
70586288pyR	Biotin-TTAATCCATGCATCCCTTCC		
70586288pyS	GCCATGGCCTTCAGTTTG		
70600816pyF	CTTCTGCCATCGACCTGTGC	122	ss666793773
70600816pyR	Biotin-GGAGAACCGGGTCACAGCAT		
70600816pyS	GGAGAGCTTCCCTCCCTC		

D. Primer information used for RT-PCR and RACE

Primer	Sequence		Purpose
PDSS2-RT-1F	GGTACCCGACGTCCTTCAT		RT-PCR
PDSS2-RT-4F	GATGCGAGGTTTGGTCGTAT		RT-PCR
PDSS2-RT-6F	CGTGGCATAGTGAACATTGG		RT-PCR
PDSS2-RT-7F	TCAAGTGCCATTGGTGATTT		RT-PCR
PDSS2-RT-8F	ATAGCGCTGAGATGCAGGAT		RT-PCR
PDSS2-RT-9F	ACTGTGCTCCTGCAGTCCTT		RT-PCR
PDSS2-RT-1R	CACAGGACTTGAGCTCACCA		RT-PCR
PDSS2-RT-2R	ATCCTGCATCTCAGCGCTAT		RT-PCR
PDSS2-RT-3R	TTCCCTGAATTTTGCCTGTC		RT-PCR
PDSS2-RT-4R	AAATCACCAATGGCACTTGA		RT-PCR
PDSS2-RT-5R	AGGACTGCAGGAGCACAGTT		RT-PCR
PDSS2-RT-8R	CTCAGAGGGAGGGAAGCTCT		RT-PCR
SOBP-RT-1F	AGGGAGACCTCCCGAAAATA		RT-PCR
SOBP-RT-2F	AATGAGCTCCTTGGCTGGTA		RT-PCR
SOBP-RT-3F	ACTCACCAGCAGGGTCAAAG		RT-PCR
SOBP-RT-4F	TCAAGCGCTATTCCCTGAGT		RT-PCR
SOBP-RT-5F	TCCCGAAAATAAAAGGAGCAGGA		RT-PCR
SOBP-RT-6F	ATGAGCTCCTTGGCTGGTATGGT		RT-PCR
SOBP-RT-7F	CGGGAAATGGACTTTCAGACTCA		RT-PCR
SOBP-RT-8F	GCTTGCCGAAGAGCATATTTCAA		RT-PCR

SOBP-RT-10F	CTGGACCATCAGCGTCTACCACT		RT-PCR
SOBP-RT-11F	AAGCCACCCAATAGCTCTTCGTC		RT-PCR
SOBP-RT-1R	CTTTGACCCTGCTGGTGAGT		RT-PCR
SOBP-RT-2R	ACTCAGGGAATAGCGCTTGA		RT-PCR
SOBP-RT-3R	TTCTTGAAGGCAAGTCTTGGA		RT-PCR
SOBP-RT-4R	AAGTTTTCGCCGTTGCTAGA		RT-PCR
SOBP-RT-5R	CTTCGTGATGGGAAGCTGCTTTA		RT-PCR
PDSS2-5R-GSP1R	TTGCATATCCTTCAGCGGACCA		5' RACE
PDSS2-5R-GSP2R	ACCAATGTTCACTATGCCACGATG		5' RACE
PDSS2-5R-GSP3R	ACGACCAAACCTCGCATCTGTAA		5' RACE
3Race-d(T)18	AACTGGAAGAATTCGCGGCCGCAGGAATTTTTTTTTTT TTTTTTT		3' RACE
3Race-R1	AACTGGAAGAATTCGCGGCC		3' RACE
SOBP-3Race-1F	GTGGTTTTGCTGCTACACTAAGAAT		3' RACE

E. Primer information used for qRT-PCR

Primer	Sequence	Length (bp)	Method
GAPDH-taqman-F	TTGTTGACCTGACCTGCCG	121	Taqman
GAPDH-taqman-R	ACAACCTGGTCCTCTGTGTATCC		
GAPDH-taqman-Probe	FAM-ACCAGCCAAGTATG-MGB		
PDSS2-taqman-F	GCTGCAATGGAACTAGCAAAG	110	Taqman
PDSS2-taqman-R	ACAAATGGCTGAAGGTCTGAA		
PDSS2-taqman-Probe	FAM-CCATGAGTCATAAGCTG-MGB		
GAPDH-qF	CGATCTGAACTACATGGTTTACATGTT	82	SYBR Green
GAPDH-qR	CCCGTTCTCAGCCTTGACA		
SOBP-qF	AATTTTCCCCAGCAGCACTA	127	SYBR Green
SOBP-qR	CGTCCCCAAAATCCAGATAC		
OSTM1-qF	CGATGTGGAAGATGCAATGAA	95	SYBR Green
OSTM1-qR	CGGCAATCACTGGGACTGTAT		
SEC63-qF	GAGGAAACAAACAGGGACTCTCA	120	SYBR Green
SEC63-qR	TTGCTGTAACTCTTGCCACTCAG		
SCML4-qF	GTGTGAGCACCTCTTCAGCAAT	124	SYBR Green
SCML4-qR	ATCCAGGTAGTCCTCAGGGATG		
BEND3-qF	ACGGAAACAGACCTCACTTGGT	100	SYBR Green
BEND3-qR	TGAATTGGAGCCctcttGTGTA		
C6orf203-qF	TCTCTGCGATATGATGTGGTCAT	101	SYBR Green
C6orf203-qR	TTTTCTCCATTCAGCCTGAGTTC		

QRSL1-qF	ATGAACGAGTCCACAGAAAGCA	145	SYBR Green
QRSL1-qR	GTCTGACTTTCTGCGCTTTGAC		
RTN4IP1-qF	AATCTGGAAGTTTGGAACAGCAG	93	SYBR Green
RTN4IP1-qR	CTAGTGCCCACTTCTCAGTGGAT		
AIM1-qF	ATTGGCATATTAGTCCCGATGG	148	SYBR Green
AIM1-qR	AGTGGTTCCCAGCACTGTGTTA		
ENSGALT00000037260-qF	GCCAGCTGAGAGACAGACAAGTT	131	SYBR Green
ENSGALT00000037260-qR	GCGCCTTAAAATTCAGCTCTACA		

F. Primer information used for transfection construct

Primer	Sequence		Purpose
BamHI-EGFP-F	cgcGGATCCATGGTGAGCAAGGGCGAGGA		eGFP
EGFP-PDSS2-R	CTCCGCCGCGGCCCCAGCGCCGCAGCAGGGCCCCCC ACATCTTGTACAGCTCGTCCATGC		
EGFP-PDSS2-F	CGCCGCCGGGATCACTCTCGGCATGGACGAGCTGTAC AAGATGTGGGGGGCCCTGCTGCG		PDSS2
XhoI-PDSS2-R	ccgCTCGAGTCAGGAGAACCGGGTCACAG		

G. Primer information used for luciferase reportor analysis

Primer	Sequence	Length (bp)	Purpose
70486623LFF	ctagctagcGGTGTGAGAACAGTGACCCCCTTG	643	Luciferase reportor
70486623LFR	ccgctcgagCCCTGGGGGCAGCCATCTT		
70486623SFF	ctagctagcAAACGCAATGCCCCGTTTTTCAC	405	Luciferase reportor
70486623SFR	ccgctcgagCCCTGGGGGCAGCCATCTT		
70486623LRF	ctagctagcGTCCCACTGTCTCCTCACAACAGG	752	Luciferase reportor
70486623LRR	ccgctcgagAGACGTGGCTGTTAGGAAGAGGTTC		
70486623SRF	ctagctagcGATCTTCTCGGCTTCCGACACC	399	Luciferase reportor
70486623SRR	ccgctcgagGGGACACGGGGAATGAATTTTG		
